# Inhibition of HDAC6 With CAY10603 Ameliorates Diabetic Kidney Disease by Suppressing NLRP3 Inflammasome

**DOI:** 10.3389/fphar.2022.938391

**Published:** 2022-07-14

**Authors:** Qing Hou, Shuyan Kan, Zhuang Wang, Jinsong Shi, Caihong Zeng, Dahai Yang, Song Jiang, Zhihong Liu

**Affiliations:** ^1^ National Clinical Research Center for Kidney Diseases, Jinling Clinical College, Southeast University School of Medicine, Nanjing, China; ^2^ National Clinical Research Center for Kidney Diseases, Jinling Hospital, Nanjing University School of Medicine, Nanjing, China; ^3^ State Key Laboratory of Bioreactor Engineering, East China University of Science and Technology, Shanghai, China

**Keywords:** connectivity map, diabetic nephropathy, HDAC6, NLRP3 inflammasome, tubular injury

## Abstract

**Background:** Diabetic nephropathy (DN) is one of the leading causes of chronic kidney disease (CKD) worldwide, tubular injury is the driving force during the pathogenesis and progression of DN. Thus, we aim to utilize the connectivity map (CMap) with renal tubulointerstitial transcriptomic profiles of biopsy-proven DN to identify novel drugs for treating DN.

**Methods:** We interrogated the CMap profile with tubulointerstitial transcriptomic data from renal biopsy-proven early- and late-stage DN patients to screen potential drugs for DN. Therapeutic effects of candidate drug were assessed in Murine model of diabetic kidney disease (STZ-induced CD-1 mice), and HK-2 cells and immortalized bone marrow-derived macrophages (iBMDMs).

**Results:** We identified CAY10603, a specific inhibitor of histone deacetylase 6 (HDAC6), as a potential drug that could significantly reverse the altered genes in the tubulointerstitial component. In DN patients and mice, upregulation of HDAC6 was mainly observed in renal tubular cells and infiltrated macrophages surrounding the diluted tubules. In both early- and late-onset diabetic mice, daily CAY10603 administration effectively alleviated renal dysfunction and reduced macrophage infiltration, tubular injury and tubulointerstitial fibrosis. Mechanistically, CAY10603 suppressed NLRP3 activation in both HK-2 cells and iBMDMs.

**Conclusion:** CAY10603 exhibited therapeutic potential for DN by suppressing NLRP3 inflammasome activation in both tubular cells and macrophages.

## Introduction

DN is a major cause of CKD in the majority of developed and developing countries (Alicic et al., 2017). For a long time, the treatment for DN has been limited to angiotensin-converting-enzyme inhibitors (ACEis) or angiotensin-receptor blockers (ARBs) (Doshi and Friedman, 2017) although sodium-glucose cotransporter 2 (SGLT2) inhibitors (Heerspink et al., 2018), including canagliflozin (Perkovic et al., 2019), dapagliflozin (Heerspink et al., 2020), and empagliflozin (Wanner et al., 2016), exhibit novel and promising benefits for improving renal function in patients with DN. Sotagliflozin, a dual inhibitor of SGLT1 and SGLT2, is able to improve kidney outcomes in patients with diabetes (Bhatt et al., 2021). Treatment with Finerenone, a nonsteroidal, selective mineralocorticoid receptor antagonist, results in lower risks of CKD progression in patients with CKD and type 2 diabetes (Bakris et al., 2020). However, new therapeutic drugs still need to be explored.

The Connectivity Map (CMap) has generated a database containing more than 1.5 million gene expression profiles from over 5,000 small-molecule compounds and exhibits the potential to identify novel drugs that reverse transcriptomic signatures for specific diseases (Lamb et al., 2006; Subramanian et al., 2017). Recently, CMap with transcriptomic profiling from proteinuric mouse glomeruli identified HDAC1 and HDAC2 as targets for proteinuric kidney disease treatment (Inoue et al., 2019). Computational drug repurposing with CMap identified vorinostat as a candidate therapy to reverse kidney disease progression in Col4a3^−/−^ mice (Williams et al., 2020). CMap analysis with 15 gene expression signatures from 11 DKD-related published independent studies identified the PLK1 inhibitor BI-2536 as a novel therapy for DKD (Zhang et al., 2021). Tubulointerstitial injury plays an important role in renal function decline and progression in DN (Ruiz-Ortega et al., 2020). In the current study, by interrogating the CMap profile with tubulointerstitial transcriptomic data from renal biopsy-proven early- and late-stage DN patients, we found that CAY10603, a specific inhibitor of HDAC6 (Sixto-López et al., 2019), significantly reversed the transcriptomic signatures in the tubulointerstitial component of DN.

HDAC6, a class II HDAC, is a cytoplasmic enzyme that plays important roles in multiple cellular processes (Miyake et al., 2016). Several studies have demonstrated that pharmacological inhibition of HDAC6 by various selective inhibitors, including tubastatin A, tubacin, ACY-738, and ACY-1215, effectively limits the progression of numerous kidney diseases (Ke et al., 2018). In a rat model of CKD, tubastatin A attenuated proteinuria, limited tubule cell death and diminished tubulointerstitial fibrosis (Brijmohan et al., 2018). In a Pkd1-conditional mouse model of ADPKD, tubacin reduced cyst growth and improved renal function (Cebotaru et al., 2016). In db/db mice, tubacin reduced albuminuria and restored renal function by suppressing autophagy and enhancing the motility of podocytes (Liang et al., 2020). In NZB/W mice, ACY-738 decreased SLE pathogenesis by inhibiting immune complex-mediated glomerulonephritis (Regna et al., 2016). In UUO mice, ACY-1215 attenuated the development of renal fibrosis by suppressing TGF-β1 and EGFR signalling (Chen et al., 2020).

CAY10603 has not yet been studied in kidney disease. We therefore investigated the effects of pharmacological inhibition of HDAC6 with CAY10603 in STZ-induced early- and late-onset diabetic CD-1 mice. CAY10603 improved renal function and halted tubular fibrosis in diabetic mice against NLRP3 inflammasome activation in both tubular cells and macrophages.

## Methods

### Human Renal Biopsy Samples

All renal biopsy proven patients (Supplementary Table S1) were from Nanjing Glomerulonephritis Registry, National Clinical Research Center for Kidney Diseases, Jinling Hospital, Nanjing, China. All patients signed a written informed consent. For tissue transcriptome analysis, gene expression profiles from 70 DN patients and 30 renal carcinoma patients with para-carcinoma renal tissue were used. 35 patients were in early stage of DN with microalbuminuria<30 mg/24 h, estimated glomerular filtration rate (eGFR) >90 ml/min 1.73 m (Doshi and Friedman, 2017); 35 patients were in late stage of DN with eGFR <60 ml/min.1.73 m (Doshi and Friedman, 2017) and proteinuria >1 g/24 h.

### Gene Expression Analysis

Renal tissue transcripts profile was performed using the Illumina HiSeqTM 2,500 platform. The gene expression Cel files are available at Sequence Read Archive (www.ncbi.nlm.nih.gov/sra/) and Gene Expression Omnibus (www.ncbi.nlm.nih.gov/geo/) under reference nos. PRJNA666231 and GSE158230, respectively.

### Connectivity Map Analysis

CMap was used to discovery potential drugs which can reverse the transcriptomic signatures in tubulointerstitial component of DN. CMap is a program for predicting potential drugs that may induce biological status encoded by specific gene expression signatures. Upregulated genes and downregulated genes (fold change >1.5, adjusted *p* value < 0.05) were used to query the CMap database. Finally, the enrichment score representing similarity was calculated, ranging from −1 to 1. The positive connectivity score indicates that drugs can induce the biological phenomena queried in human cell lines. Conversely, a negative connectivity score indicates that the drug reverses the requested biological characteristics and has potential therapeutic value. The connectivity scores (*p* < 0.05) for the various instances were filtered out.

### Reagents

LPS (tlrl-pb5lps, Invivogen), ATP (tlrl-atpl, Invivogen), Nigericin (N1495, Invitrogen), CAY10603 (HY-18613, MedChemExpress), Streptozotocin (S1312, Selleck), Mounting medium with DAPI (ab104139, Abcam), and Mounting Medium for IHC (ab64230, Abcam).

### Antibodies

HDAC6 (Cell Signaling Technology Cat# 7612, RRID:AB_10889735; 1:1000 in WB, 1:100 in IHC), NLRP3 (Cell Signaling Technology Cat# 13158, RRID:AB_2798134; 1:1000 in WB), NLRP3 (AdipoGen Cat# AG-20B-0014, RRID:AB_2490202; 1:1000 in WB), Caspase-1 (Proteintech Cat# 22915-1-AP, RRID:AB_2876874; 1:3000 in WB), Caspase-1 (AdipoGen Cat# AG-20B-0042, RRID:AB_2490248; 1: 1000 in WB), Cleaved N-terminal GSDMD (Abcam Cat# ab215203, RRID not available; 1:500 in WB), ASC (Santa Cruz Biotechnology Cat# sc-514414, RRID:AB_2737351; 1:500 in WB), α-Tubulin (Proteintech Cat# 11224-1-AP, RRID:AB_2210206; 1:5000 in WB), β-Tubulin (Proteintech Cat# 10068-1-AP, RRID:AB_2303998; 1:5000 in WB), acetylated α-Tubulin (Lys40) (Proteintech Cat# 66200-1-Ig, RRID:AB_2722562; 1:1000 in WB), α-SMA (Proteintech Cat# 14395-1-AP, RRID:AB_2223009; 1:2000 in WB, 1:300 in IHC), Col1a (Cell Signaling Technology Cat# 72026, RRID:AB_2904565; 1:2000 in WB, 1:300 in IHC), Goat anti-mouse IgG (H + L), HRP conjugate (SA00001-1, Proteintech), Goat anti-rabbit IgG (H + L), HRP conjugate (SA00001-2, Proteintech), Donkey anti-rabbit IgG (H + L), Alexa Fluor 488 (A-21206, ThermoFisher), Donkey anti-mouse IgG (H + L), Alexa Fluor Plus 555 (A32773, ThermoFisher).

### STZ-Induced Diabetic Kidney Disease in Mice

8-week-old male CD-1 mice (Charles River, Beijing) received intraperitoneal a single injection of STZ (150 mg/kg body weight) dissolved in sodium citrate buffer (pH 4.5), control groups of mice were injected with the same volume of sodium citrate buffer. 1 week after STZ injection, successful induction of diabetes was confirmed by tail vein blood glucose level >16 mM CAY10603 (5 mg/kg body weight) and the same volume vehicle were intraperitoneal injected daily from 12 weeks after STZ injection for 3 weeks, mice were sacrificed, blood samples and kidney tissues were collected. 4–6 mice per cage were housed under standard conditions. Sample numbers in each experimental group were indicated in figures.

### Kidney Function Analysis and Histology

Serum was prepared by centrifugation of the blood at 1000 g for 20 min at 4°C and stored at −80°C for further analysis including LDH, Creatinine, BUN. Urine albumin (Albumin ELISA Kit, GeneTex) and creatinine (QuantiChrom™ Creatinine Assay Kit, BioAssay Systems) were tested according to the manufacturer’s instructions. Mouse kidneys were fixed in 4% PFA, embedded in paraffin, and cut into 2.5-µm sections. Sections were stained with Hematoxylin and Eosin, and Periodic acid-Schiff, Masson.

### Immunohistochemistry and Immunofluorescence Staining

Paraffin-embedded kidney sections from human and mouse were deparaffinized, blocked with 10% BSA in PBS for 30 min at room temperature (RT) and then incubated with primary antibodies as indicated at 4°C overnight. The next day, after five times washes with PBS, incubate with secondary antibodies at RT for 1 h. For IHC staining, diaminobenzidine (DAB) color reaction was kept with a fixed exposure time for all experiments among the groups. For IF staining, indicated secondary antibodies were incubated for 2 h at RT in dark and then mounted with DAPI for confocal microscope imaging (Zeiss LSM900, Germany).

### Immortalized Mouse Bone Marrow–Derived Macrophages Culture and NLRP3-Engaged Pyroptosis Induction

iBMDMs were cultured in IMDM supplemented with 10% (v/v) FBS, penicillin (100 U/mL) and streptomycin (100 μg/ml) at 37°C incubator adding 5% CO_2_. The cells were digested with 0.25% trypsin until the confluence reached 80%. iBMDMs were seeded into 12-well plate at a density of 5 × 10^5^ CFU/ml. After overnight culture, the cells were primed with 250 ng/ml LPS resolved in Opti-MEM for 4 h. Then, the cells were stimulated with 2.5 mM ATP or 20 μM nigericin for 1 h. For HDAC6 inhibition assay, the inhibitor CAY10603 was pre-incubated with the cell for 1 h before LPS priming and continuously added during the subsequent treatment. The phenotypes of pyroptosis in different treatment groups were visualized by microscopy imaging (DMI3000B, Leica).

### Cytotoxicity and IL-1β Secretion Assay

The cytotoxicity of iBMDMs after stimulation was determined by using a CytoTox 96 Assay Kit (G1780, Promega) according to the manufacturer’s instructions. The quantitative values of cell death were measured by a microplate reader (SpectraMax M5) at OD_490_ nm. For IL-1β secretion assay, the supernatant after stimulation and serum were collected and measured using an IL-1 beta Mouse ELISA Kit (BMS6002, Invitrogen) according to the manufacturer’s instructions.

### HK-2 Cell Culture and Treatments

The human proximal tubular epithelial cell line (HK-2) was purchased from the American Type Culture Collection (ATCC). Cells were cultured in Dulbecco’s Modified Eagle’s Medium/Nutrient Ham’s Mixture F-12 (DMEM/F12, STEMCELL) supplemented with 10% fatal bovine serum (FBS), penicillin (100 U/ml) plus streptomycin (0.1 mg/ml). Culture conditions were maintained at 37°C in a humidified atmosphere at 5% CO_2_. For HDAC6 inhibition assay, CAY10603 was pre-incubated with the cells for 4 h before TGF-β treatment.

### Immunoblot Analysis

Cells were lysed using M-PER™ Mammalian Protein Extraction Reagent (78505, Thermo Scientific™). The proteins were run in 4%–12% FUTURE™ PAGE gels (FUTURE BioTec, Nanjing, China) and then transferred to PVDF membranes (Millipore Merck). After blocking with NcmBlot blocking buffer at room temperature for 10 min, blots were incubated with primary antibodies (diluted with NCM Universal Antibody Dilution) overnight at 4°C.

### Statistical Analysis

All values are presented as the mean ± SEM, comparisons between two groups were performed using two-tailed unpaired *t* test, comparisons between multiple groups were performed using Two-way ANOVA. GraphPad Prism version 9.0 was used for statistical analysis, *p* value <0.05 was considered to be statistically significantly.

### Study Approval

All experimental protocols for animal studies were approved by the Institutional Animal Care and Use Committee of Jinling Hospital, Nanjing University School of Medicine (Document No. 2021DZGKJDWLS-00153). The protocol for the use of human samples was approved by the Human Subjects Committee of Jinling Hospital, Nanjing University School of Medicine (2013KLY-013-01), and a signed consent form was obtained from each patient and control donor.

### Role of Funders

The funders had no role in the study design, data collection, data analysis, interpretation of results, or writing of report.

## Results

### CMap Analysis With Tubulointerstitial Transcriptomic Profiles From DN Patients

**FIGURE 1:**
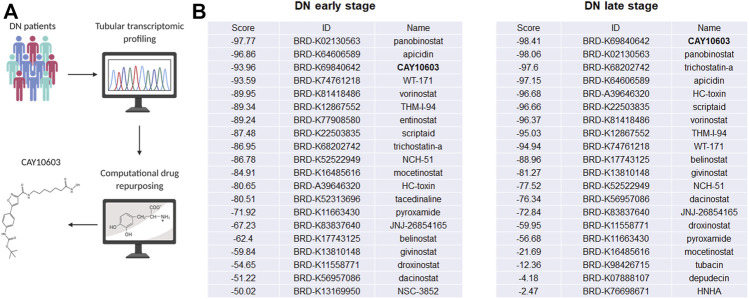
Tubular transcriptomics from a biopsy-proven DN patient-based drug repurposing approach to identify potential drugs for treatment. **(A)** Schematic view of work flow. **(B)** The top 20 HDAC inhibitors are sorted in each stage, and CAY10603 is highlighted in black.

To predict potential small molecule compounds to reverse the tubulointerstitial transcriptomic expression profiles of DN patients, the CMap database was queried with the top 150 upregulated and 150 downregulated genes (fold change >1.5, adjusted *p* value < 0.05; Supplementary Tables S2, S3) extracted from tubulointerstitial transcriptomic expression profiles of early- and late-stage DN patients, respectively (Figure 1A). The queries generated the list of small molecule compounds with different enrichment scores. A negative connectivity score indicates that the drug reverses the requested biological characteristics and has potential therapeutic value. We evaluated the top lists of small molecule compounds that had the highest connectivity score that were predicted to reverse the input genes in early- and late-stage DN patients. It is worth noting that HDAC inhibitors appeared most often in the top 100 predicted small molecule drugs, as listed in Supplementary Table S4. Among the candidate HDAC inhibitors (Figure 1B), CAY10603 attracted our attention because it was one of the few top drugs in both lists; in addition, it has an extremely lower half maximal inhibitory concentration (IC50) and higher selectivity against HDAC6 as comparing with tubastatin A, tubacin, ACY-738 and ACY-1215, >200-fold over other HDACs.

### Increased HDAC6 Expression Is Associated With Tubular Injury

**FIGURE 2:**
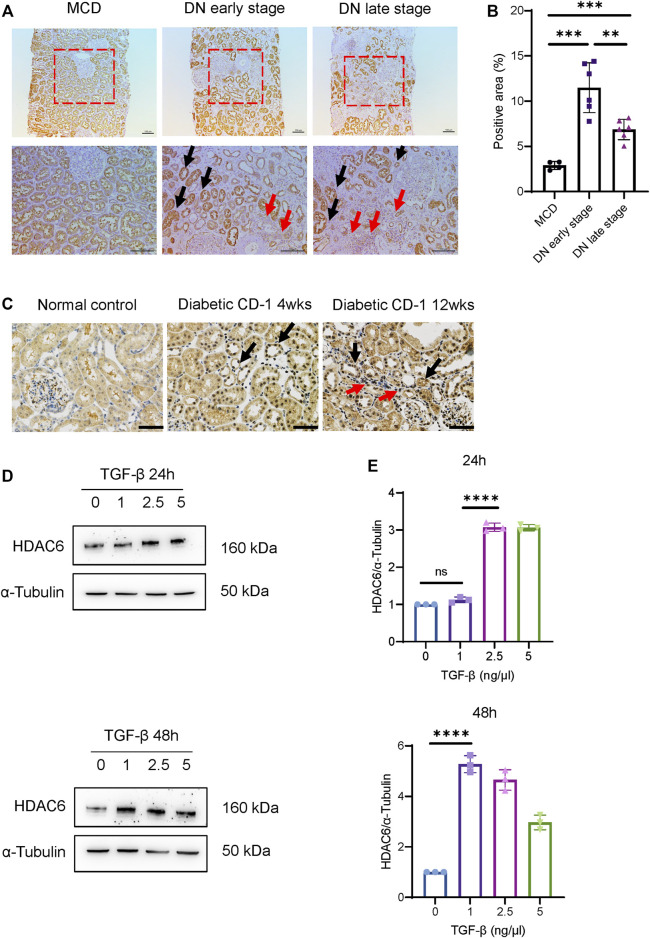
HDAC6 upregulation is associated with tubular injury. **(A)** IHC staining images of HDAC6 expression distribution in renal tissues from DN patients and MCD. HDAC6 is increased in tubular cells (black arrows) and intrarenal macrophages (red arrows). Scale bar, 100 µm. **(B)** Quantification of HDAC6 IHC staining. *n* = 4 in the healthy control group, *n* = 6 in the early-stage DN group, *n* = 6 in the late-stage DN group. **(C)** IHC staining images of HDAC6 expression distribution in renal tissues from diabetic CD-1 mice and normal control. HDAC6 is increased in tubular cells (black arrows) and tubulointerstitial infiltrated cells (red arrows). Scale bar, 50 µm. **(D)** Representative western blot analysis and **(E)** quantification of HDAC6 levels in HK-2 cells treated with TGF-β for 24 h or 48 h at different dosages as indicated. **p < 0.01; ***p < 0.001; ****p < 0.0001. Unpaired two-tailed *t* test.

We next determined the expression pattern of HDAC6 in the kidney tissue of DN patients. Compared to minimal changed disease (MCD) with mild tubular injury, the immunohistochemistry (IHC) staining results (Figures 2A,B) showed that renal tubular expression of HDAC6 was significantly increased in the early stage of DN and decreased slightly in the late stage of DN. We observed that tubulointerstitial expression of HDAC6 was clearly upregulated around diluted tubules in the late stage of DN, indicating that tubulointerstitial HDAC6 correlated with tubular injury. Infiltration of immune cells, predominantly macrophages, was commonly observed in the interstitium of patients’ kidney tissues at all stages of DN (Calle and Hotter, 2020); thus, we confirmed that increased expression of HDAC6 was mainly in CD68^+^ macrophages (Supplementary Figure S1).

IHC staining also confirmed that HDAC6 was upregulated in tubular epithelial cells and tubulointerstitial infiltrated cells in kidneys of diabetic CD-1 mice (Figure 2C **)**. In HK-2 cells, HDAC6 was significantly induced by 2.5 and 5 ng/μl TGF-β stimulation after 24 h and induced by 1, 2.5 and 5 ng/μl TGF-β stimulation after 48 h (Figures 2D,E). The data indicated that HDAC6 contributed to the pathogenesis of renal interstitial damage.

### CAY10603 Improves Renal Function and Tubulointerstitial Fibrosis in both Early- and Late-Onset Diabetic Mice

To evaluate the inhibitory effects of CAY10603 on the progression of early- and late-stage DKD *in vivo*, STZ-induced diabetic CD1 male mice (Sugimoto et al., 2007; Li et al., 2020) were used, which exhibited heavy proteinuria and mild tubular injury at 4 weeks and severe tubular injury and fibrosis at 12 weeks after the induction of diabetes from STZ injection. IHC staining demonstrated CD-1 mice had higher expression levels of HDAC6 in renal tubular cells at 4 and 12 weeks after the induction of diabetes as comparing to normal mice. We also observed the expression of HDAC6 in infiltrated cells in the interstitium of diabetic kidneys.

**FIGURE 3:**
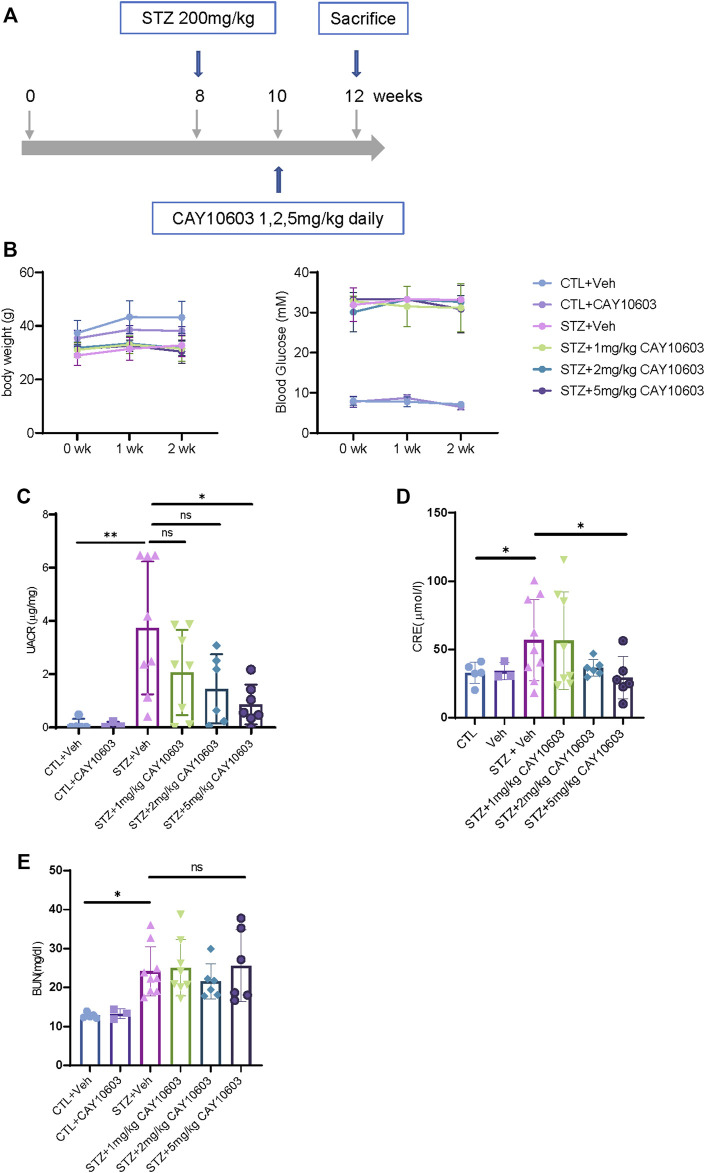
CAY10603 improves kidney function in the early stage of diabetic mice. **(A)** Schematic diagram of experiment design. **(B)** body weight and blood glucose in different groups and time point as indicated. **(C)** Urine albumin to creatinine ratio in different groups as indicated. **(D)** Serum creatinine and **(E)** serum BUN in different groups as indicated. *p < 0.05; **p < 0.01. Two-way ANOVA.

**FIGURE 4:**
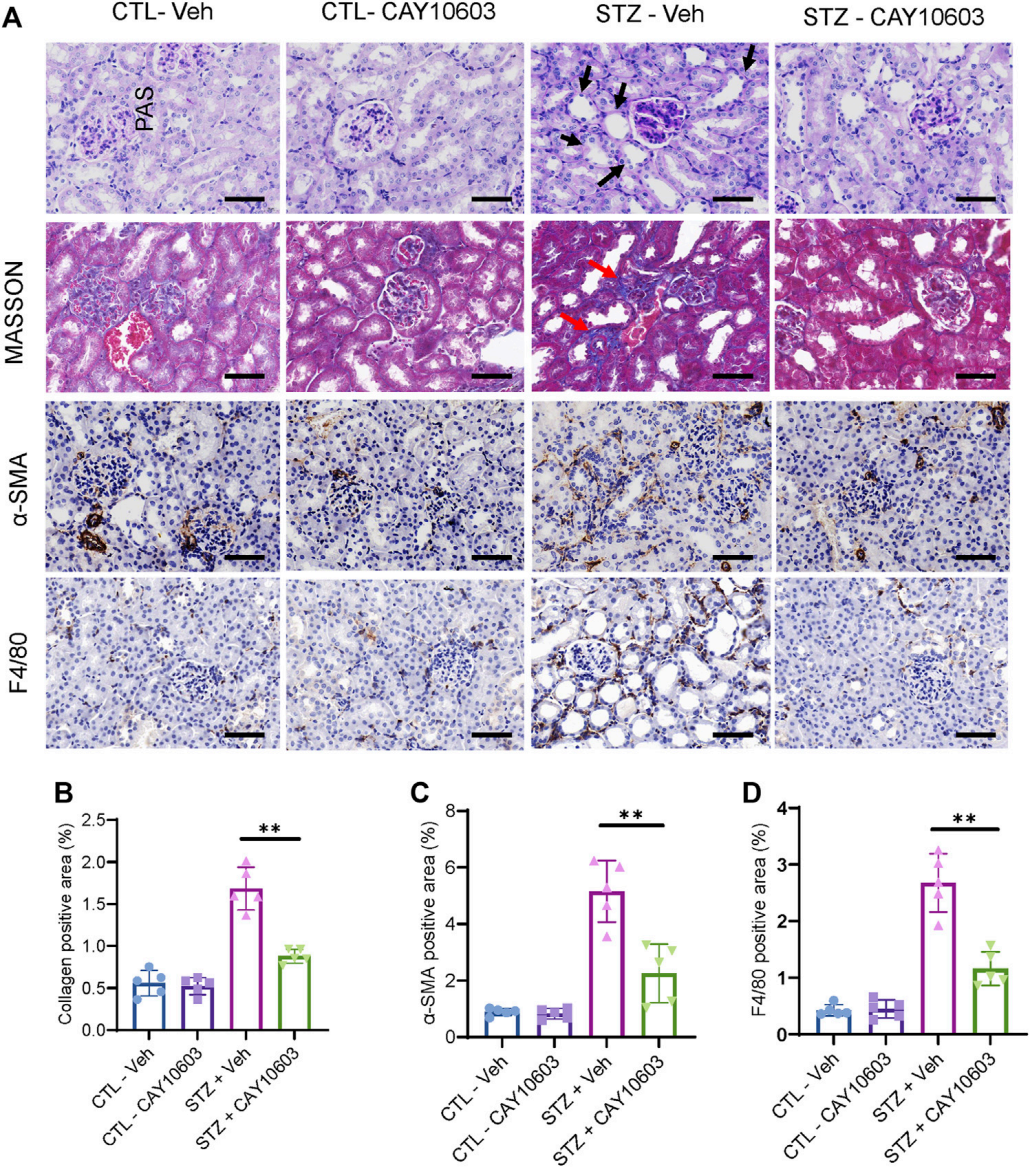
CAY10603 improves kidney histology in the early stage of diabetic mice. **(A)** Representative images of PAS staining, Masson staining, and IHC staining of α-SMA and F4/80. **(B)** Quantification of the positive area for Masson staining. **(C)** Quantification of the positive area for α-SMA IHC staining. **(D)** Quantification of the positive area for F4/80 IHC staining. Scale bar, 100 µm. Two-way ANOVA. CTL, control; Veh, vehicle.

Two weeks after the induction of diabetes, mice were randomized into either control groups or treatment groups. The mice in the treatment groups received CAY10603 at 1, 2 and 5 mg/kg body weight daily by intraperitoneal injection for 2 weeks (Figure 3A), and the mice in the control group received the same volume of vehicle by intraperitoneal injection. Compared with 1 and 2 mg/kg CAY10603, 5 mg/kg CAY10603 significantly reduced the uric albuminuria-to-creatinine (UACR) ratio (Figure 3C) and serum creatinine (Figure 3D) but did not alter serum BUN (Figure 3E), body weight (Figure 3B) or blood glucose. Consistently, histological analysis (Figure 4A) displayed great attenuation in tubular injury (Black arrows) and tubulointerstitial fibrosis (Red arrows) (Figure 4B), reduced tubulointerstitial α-SMA expression (Figure 4C), and infiltration of F4/80^+^ macrophages (Figure 4D).

**FIGURE 5:**
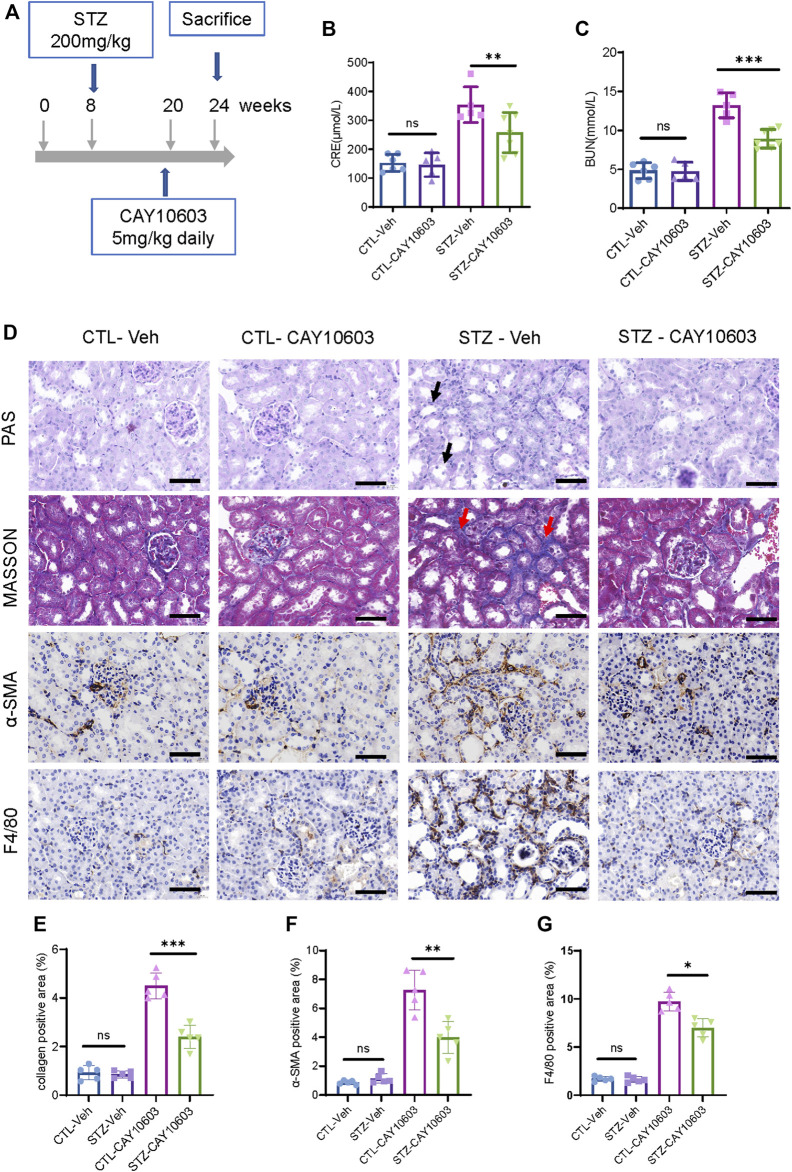
CAY10603 delays kidney function decline in the late stage of diabetic mice. **(A)** Schematic diagram of experimental design. **(B)** Serum creatinine and **(C)** serum BUN in different groups as indicated. **(D)** Representative images of PAS staining, Masson staining, and IHC staining of α-SMA and F4/80. **(E)** Quantification of the positive area for Masson staining. **(F)** Quantification of the positive area for α-SMA IHC staining. **(G)** Quantification of the positive area for F4/80 IHC staining. *p < 0.05; **p < 0.01; ***p < 0.001. Scale bar, 100 µm. Two-way ANOVA. CTL, control; Veh, vehicle.

Furthermore, diabetic mice were treated with 5 mg/kg CAY10603 starting at 20 weeks (Figure 5A). After 4 weeks, diabetic mice that received 5 mg/kg CAY10603 exhibited lower serum creatinine (Figure 5B) and BUN (Figure 5C) than diabetic mice in the vehicle group. As expected, histological examination (Figure 5D) showed that CAY10603 ameliorated tubular injury and tubulointerstitial fibrosis (Figure 5E) and reduced tubulointerstitial α-SMA expression (Figure 5F) and F4/80^+^ macrophage infiltration (Figure 5G). Taken together, these results suggested that CAY10603 treatment is effective in improving renal function in both experimental mouse models of early- and late-stage diabetic kidney disease.

### Inhibitory Effects of CAY10603 on NLRP3 *in vitro* Renal Tubular Cells

**FIGURE 6:**
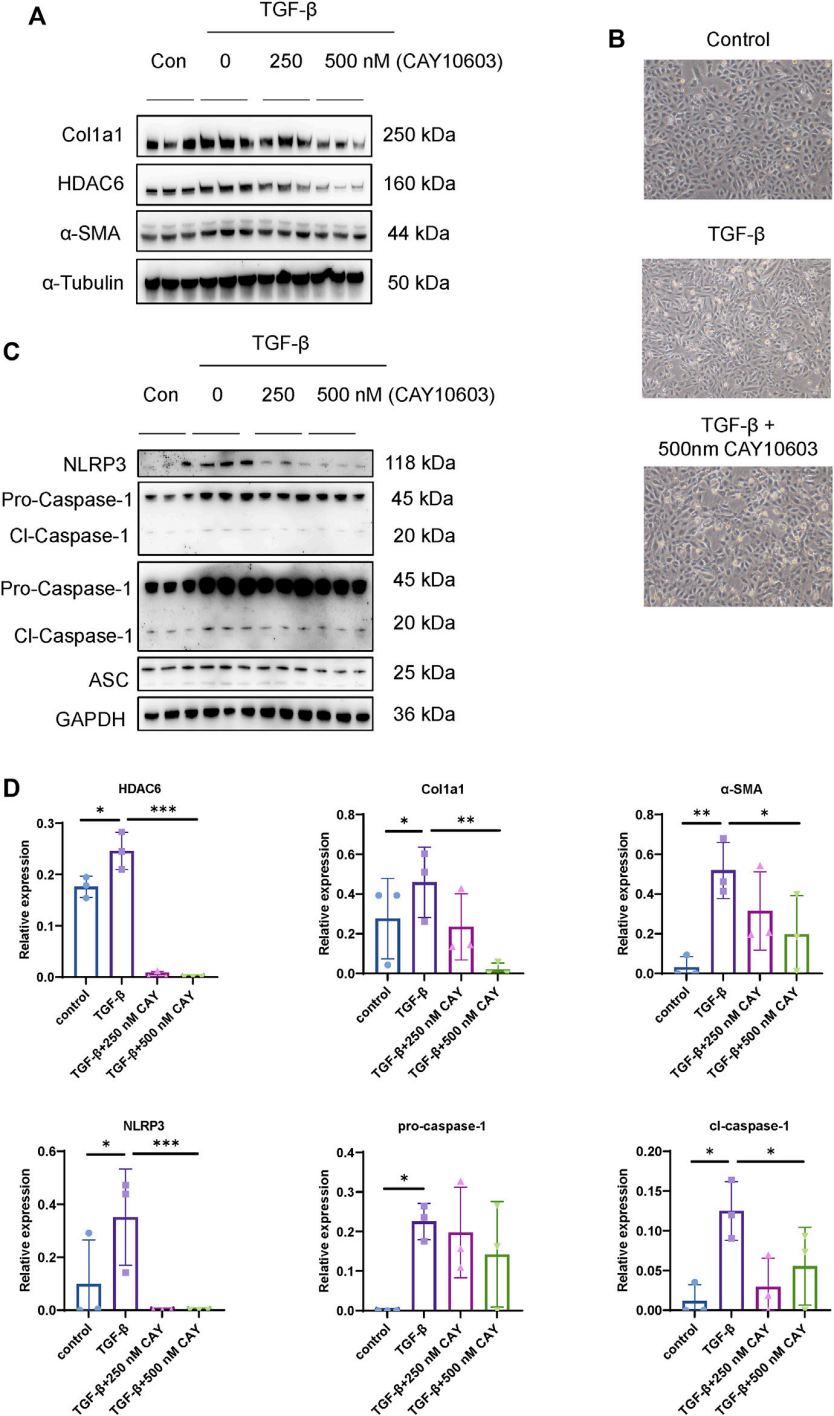
HDAC6 inhibition reduced NLRP3 activation in renal tubular cells. **(A)** Representative western blot analysis of HDAC6, Col1a, and α-SMA levels in HK-2 cells treated with TGF-β with or without CAY10603 treatment. **(B)** Representative image of the morphology of HK-2 cells treated with TGF-β with or without CAY10603 treatment. **(C)** Representative western blot analysis of NLRP3, ASC, and Caspase-1 levels in HK-2 cells treated with TGF-β with or without CAY10603 treatment. **(D)** Quantification of HDAC6, Col1a1, α-SMA, NLRP3, pro-Caspase-1, and Clevaed-Caspase-1. *p < 0.05; **p < 0.01; ***p < 0.001.

NLRP3 activation promotes renal tubular injury and fibrosis in DN (Tang and Yiu, 2020), and HDAC6 mediates an aggresome-like mechanism for NLRP3 inflammasome activation (Magupalli et al., 2020), but their relationships to tubular injury in DN are unknown. First of all, we investigated the inhibitory effect on HDAC6 expression by CAY10603, in HK-2 cells, as shown in Figure 6A, CAY10603 exhibited the inhibitory effect on HDAC6 expression in a dose-dependent manner as indicated, meanwhile, CAY10603 treatment prevented TGF-β-induced Col1a1 and α-SMA expression, as well as fibrotic morphological changes (Figure 6B). Next, we investigated the inhibitory effect on NLRP3 activation by CAY10603, TGF-β-induced activation of NLRP3, Caspase-1, and ASC were significantly suppressed by CAY1060 (Figures 6C,D). These observations revealed that CAY10603 treatment suppressed NLRP3 activation and col1a1 induction in HK-2 cells.

### HDAC6 Inhibition With CAY10603 Alleviates NLRP3 Activation in iBMDMs

**FIGURE 7:**
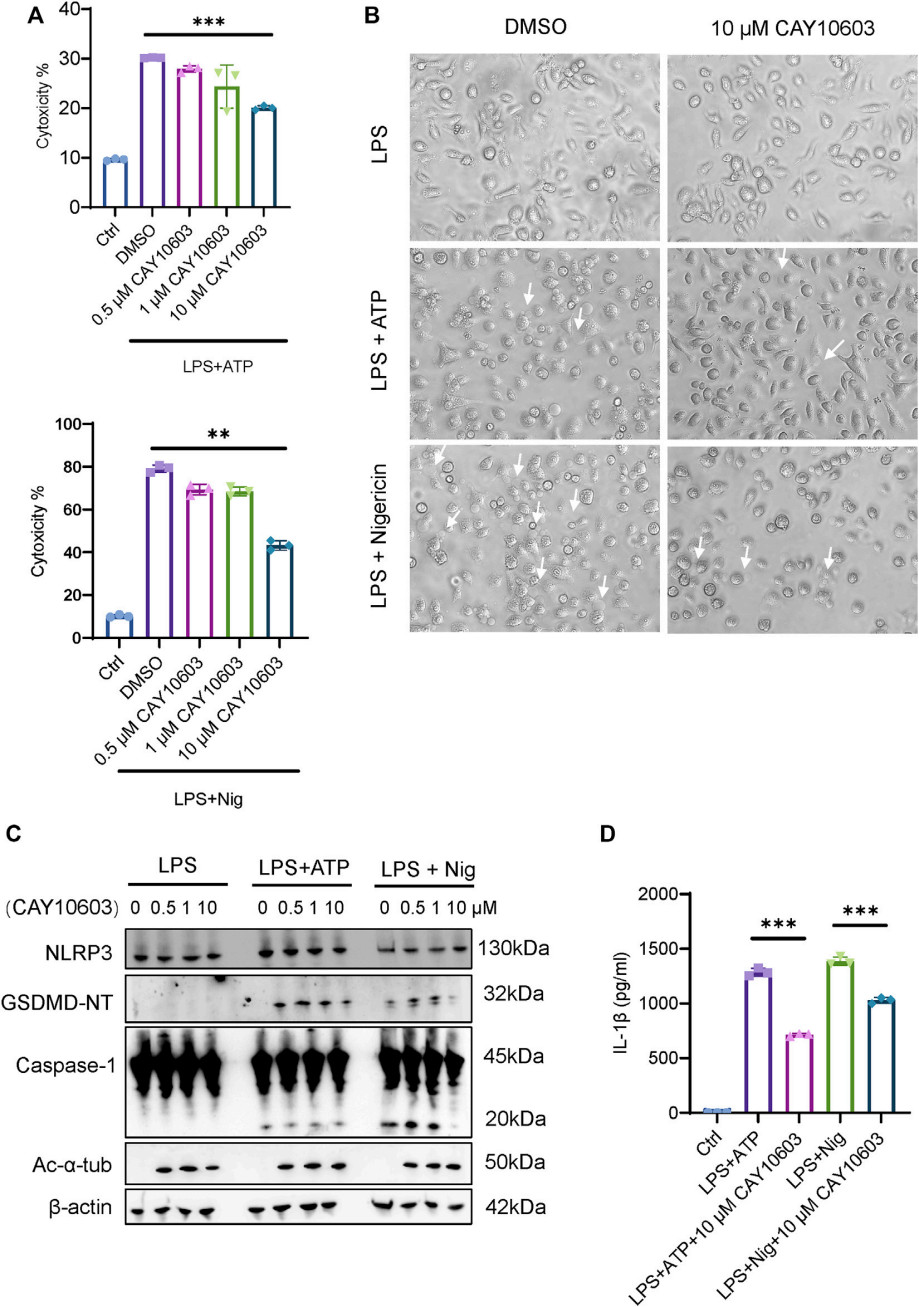
HDAC6 inhibition suppresses NLRP3 activation in macrophages. **(A)** Supernatants from the indicated iBMDMs were analysed for cell death, as measured by lactate dehydrogenase (LDH) release. **(B)** Images were taken after LPS + ATP/Nig challenge with or without CAY10603 pretreatment. Arrows indicate cells exhibiting pyroptotic-like features. **(C)** Representative western blot analysis of HDAC6, NLRP3, GSDMD-NT, Caspase-1, and Ac-α-Tub levels in iBMDMs after LPS + ATP/Nig challenge with or without CAY10603 treatment. **(D)** Supernatants from the indicated iBMDMs were analysed for IL-1β. **p < 0.01; ***p < 0.001. Two-way ANOVA.

To further investigate the effects of HDAC6 inhibition during NLRP3 activation in mouse iBMDMs, we applied an LPS-ATP/Nig stimulation method to induce NLRP3. We found that treatment with the HDAC6 inhibitor CAY10603 significantly decreased the cytotoxicity induced by LPS-ATP/Nig stimulation in a dose-dependent manner (Figure 7A), and the cells showed fewer pyroptosis phenotypes, such as cell membrane swelling and bubbling (Figure 7B). Moreover, Caspase-1 activation and GSDMD cleavage were also significantly lower when with 10 μM CAY10603 treatment, although NLRP3 expression showed little difference (Figure 7C). In addition, we also detected the expression of acetylated α-tubulin (Ac-α-tub) and verified that the inhibition was valid. IL-1β secretion in the supernatant also decreased after CAY10603 treatment (Figure 7D). These results suggest that the inhibition of HDAC6 with CAY10603 could effectively alleviate NLRP3-induced pyroptosis in macrophages.

## Discussion

Renal tubular injury is an accelerating factor that drives the process of DN (Liu et al., 2018), and targeting renal tubular cells is beneficial for developing therapeutics to delay the progression of DN (Grgic et al., 2012; Mori et al., 2021). We previously evaluated tubulointerstitial transcriptional profiles in patients with renal biopsy-proven DN. In the current work, we present the discovery of a highly selective HDAC6 inhibitor, CAY10603, as a potential therapeutic drug based on regulated genes in the tubulointerstitial component of DN patients, through use of the CMap database. In DN patients, HDAC6 is upregulated in tubular cells and intrarenal macrophages, correlating with the severity of tubular damage. *In vivo*, CAY10603 effectively alleviated renal dysfunction in both early- and late-stage diabetic mice. *In vitro*, CAY10603 suppressed NLRP3 activation and col1a1 induction in HK-2 cells and NLRP3 activation and GSDMD-induced pyroptosis in iBMDMs.

HDACs have been implicated in the pathogenesis of DN (Lee et al., 2007; Hadden and Advani, 2018). HDAC2 plays a critical role in the development of ECM accumulation and EMT in diabetic kidney (Du et al., 2020). HDAC4 contributes to podocyte injury and links renal injury to autophagy in DN, and *in vivo* gene silencing of HDAC4 reduces podocyte injury and proteinuria in diabetic rats (Wang et al., 2014). Cyclo-RGD truncated polymeric nanoconstructs with dendrimeric templates for targeted HDAC4 gene silencing inhibited the progression of renal fibrosis in an STZ-induced DN model (Raval et al., 2021). Silencing of HDAC9 attenuates glomerulosclerosis, inflammatory cytokine release, podocyte apoptosis and injury in db/db mice (Liu et al., 2016). Daily treatment of diabetic rats with vorinostat for 4 weeks blunted renal growth and glomerular hypertrophy by downregulating EGFR (Gilbert et al., 2011). Long-term administration of vorinostat attenuates renal injury in STZ-induced eNOS^−/−^mice through an endothelial nitric oxide synthase-dependent mechanism (Advani et al., 2011). Inhibiting HDAC6 by tubacin protects kidney function by modulating podocyte autophagy and motility in db/db mice (Komada and Muruve, 2019). Thus, HDAC inhibitors have been recognized as potential agents for treating DN.

CAY10603 has not been studied in kidney disease. First, we tested the effective doses. Based on UACR and creatine results, 5 mg/kg CAY10603 exhibited better effects. Subsequently, daily treatment with CAY10603 for 4 weeks effectively halted the progression of diabetic kidney injury. It is noteworthy that CAY10603 has no impact on blood glucose, indicating that the renal protection of CAY10603 is independent of blood glucose control. Combination treatment with hypoglycaemic drugs to protect against DN is worthy of consideration.

HDAC6 is a microtubule-associated deacetylase and a component of the aggresome and mediates an aggresome-like mechanism for NLRP3 inflammasome activation (Magupalli et al., 2020). The NLRP3 inflammasome not only amplifies renal inflammation but also promotes renal fibrosis, so NLRP3 inflammasome activation contributes significantly to the pathogenesis and progression of DN (Shahzad et al., 2016; Chi et al., 2017; Andrade-Oliveira et al., 2019; Wu et al., 2021). In patients with DN, the expression of NLRP3, CASP1 and IL-1β was upregulated in renal biopsy samples, and these components were also upregulated in the kidneys of diabetic mice (Shahzad et al., 2015; Han et al., 2018). Both renal resident and bone marrow-derived NLRP3 mediate inflammatory processes in DN (Son et al., 2017; Xu et al., 2021). Pharmacological inhibition or genetic knockout of HDAC6 markedly decreased NLRP3 inflammasome activation (Magupalli et al., 2020). Targeting HDAC6 attenuates nicotine-induced macrophage pyroptosis via the NF-kappaB/NLRP3 pathway in atherosclerosis (Son et al., 2017). HDAC6 regulates the activation of M1 macrophages by downregulating NLRP3 expression during acute liver failure (Chen et al., 2021). Inhibition of HDAC6 attenuates the NLRP3 inflammatory response and protects dopaminergic neurons in experimental models of Parkinson’s disease (Yan et al., 2020). Although HDAC6 regulates NLRP3 activation, the relationship in renal tubular cells is still unknown. To address this question, we challenged HK-2 cells with TGF-β and high glucose (Supplementary Figure S2) and found that HDAC6 positively correlated with NLRP3 expression, while HDAC6 inhibition with CAY10603 dramatically blocked NLRP3 activation and fibrosis. We also demonstrated that CAY10603 was effective in suppressing LPS-induced NLRP3 activation and subsequent GSDMD-mediated pyroptosis in iBMDMs.

However, there are two limitations to our study. First, the CMap database is generated from various cell types but not kidney cells challenged with chemical drugs, and the relevance to DN is a matter of concern. Second, due to the lack of mouse models with type 2 diabetes and severe interstitial fibrosis, we used an STZ-induced CD-1 diabetic mouse model with type 1 diabetes and severe kidney injury to elevate the pharmacological inhibitory effects of CAY10603.

In summary, we investigated the connectivity map (CMap) with tubulointerstitial transcriptomic profiles of renal biopsy-proven DN patients and identified CAY10603, a specific inhibitor of HDAC6, as a potential drug for treating kidney tubular damage. Mechanistically, CAY10603 inhibited NLRP3 activation and GSDMD pyroptosis in renal tubular cells and iBMDMs. Altogether, these results indicate that CAY10603 exhibits therapeutic potential for diabetic kidney injury.

## Data Availability

The datasets presented in this study can be found in online repositories. The names of the repository/repositories and accession number(s) can be found in the article/Supplementary Material.
