# Supplementary material for: Inhibition of HDAC6 With CAY10603 Ameliorates Diabetic Kidney Disease by Suppressing NLRP3 Inflammasome
Source: Front Pharmacol. 2022 Jul 14;13:938391. doi: 10.3389/fphar.2022.938391 (PMC9332914; doi:10.3389/fphar.2022.938391)
Supplement: Supplementary file 1 [file Table1.DOCX]

**Supplementary Table 1: Clinical characteristics at the time of renal biopsy**

| Gender | Age  (yrs) | Serum Creatinine (mg/dl) | 24 h proteinuria (g/24h) | eGFR  (ml/min.1.73m^2^) | Diagnosis | tubulointerstitial damage (%) |
| --- | --- | --- | --- | --- | --- | --- |
| Female | 38 | 0.48 | 0.47 | 125 | MCD | 5 |
| Male | 21 | 0.77 | 3.03 | 147 | MCD | 5 |
| Male | 29 | 0.56 | 4.12 | 140 | MCD | 5 |
| Male | 9 | 0.36 | 3.65 | 112 | MCD | 5 |
| Male | 42 | 0.96 | 1.77 | 85 | DN | 20 |
| Male | 32 | 1.3 | 2.3 | 72 | DN | 30 |
| Male | 52 | 1.23 | 1.22 | 67 | DN | 5 |
| Female | 25 | 1.13 | 3.06 | 68 | DN | 30 |
| Female | 51 | 0.67 | 2.09 | 102 | DN | 15 |
| male | 62 | 1.21 | 2.35 | 64 | DN | 30 |
| Male | 44 | 3.73 | 10.72 | 19 | DN | 60 |
| Female | 52 | 1.44 | 2.89 | 42 | DN | 50 |
| Female | 49 | 2.21 | 3.14 | 26 | DN | 70 |
| Male | 25 | 1.6 | 1.38 | 49 | DN | 30 |
| Male | 33 | 2.53 | 2.17 | 32 | DN | 40 |
| Male | 56 | 1.91 | 8.13 | 38 | DN | 50 |

**MCD, minimal change disease; DN, diabetic nephropathy.**
