# Supplementary figures and images for: Inhibition of HDAC6 With CAY10603 Ameliorates Diabetic Kidney Disease by Suppressing NLRP3 Inflammasome
Source: Front Pharmacol. 2022 Jul 14;13:938391. doi: 10.3389/fphar.2022.938391 (PMC9332914; doi:10.3389/fphar.2022.938391)

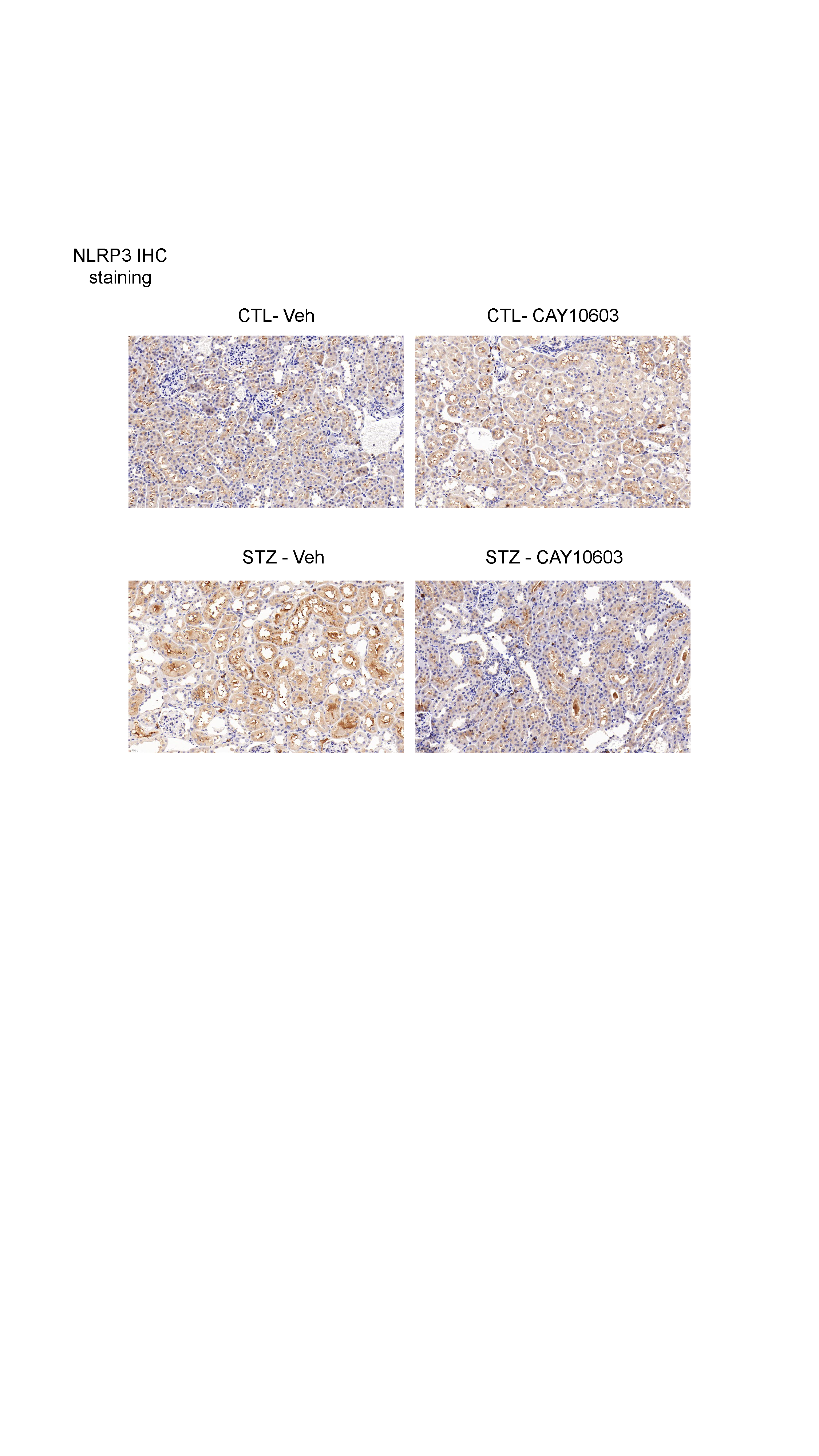

Supplement: Supplementary file 2 [file Image3.TIF]

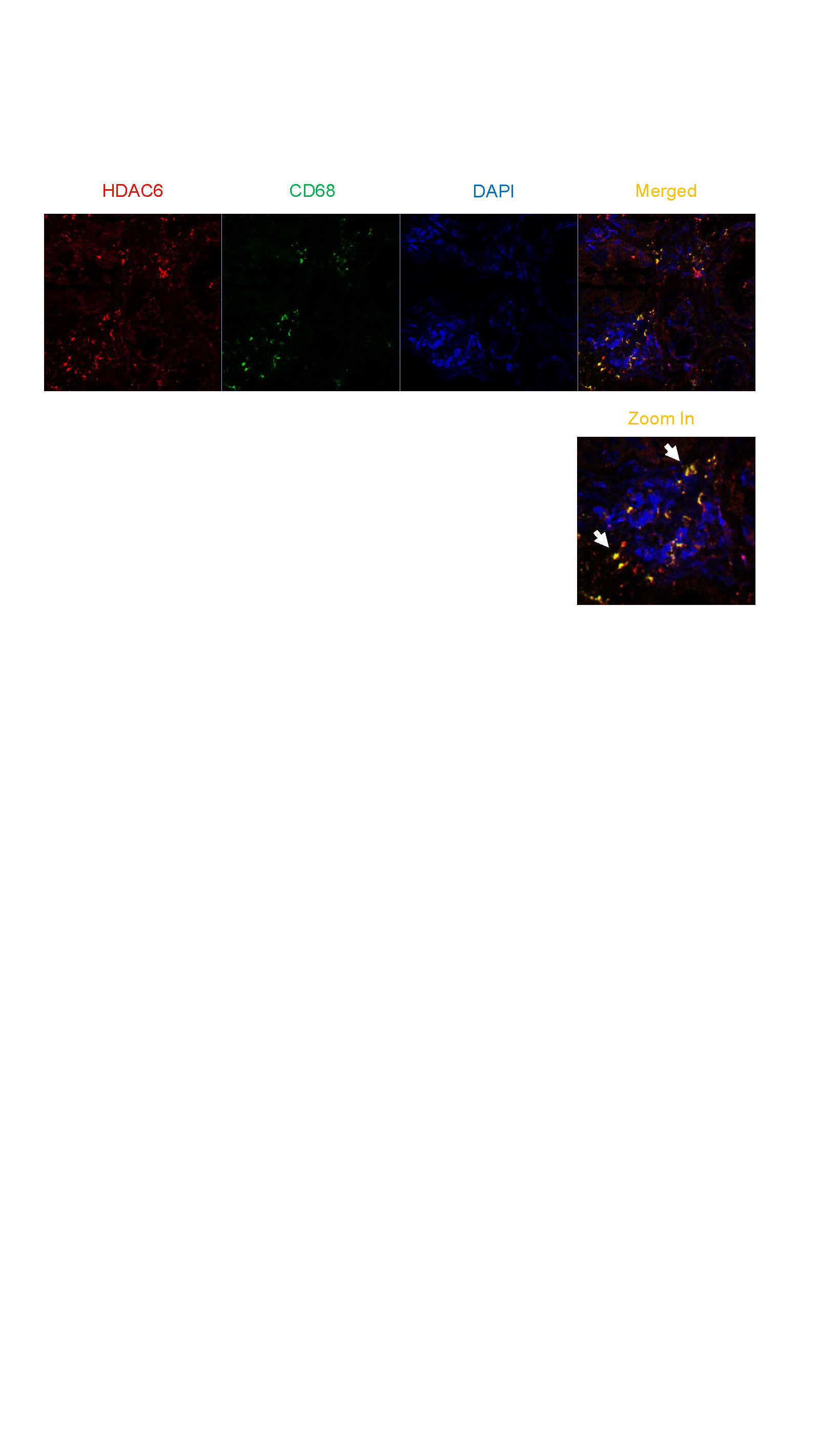

Supplement: Supplementary file 4 [file Image1.TIF]
