# Supplementary material for: Inhibition of HDAC6 With CAY10603 Ameliorates Diabetic Kidney Disease by Suppressing NLRP3 Inflammasome
Source: Front Pharmacol. 2022 Jul 14;13:938391. doi: 10.3389/fphar.2022.938391 (PMC9332914; doi:10.3389/fphar.2022.938391)
Supplement: Supplementary file 5 [file Table2.DOCX]

**Supplementary Table 2：The top 150 upregulated genes and top 150 down-regulated genes in late stage DN compared with healthy controls (fold change>1.5, p value <0.05).**

| gene_name | FoldChange（log2） | padj |
| --- | --- | --- |
| RPS24 | 1.317596 | 1.5E-162 |
| AC116533.1 | 7.339165 | 3.3E-120 |
| SET | 0.953941 | 1.6E-101 |
| SMARCE1 | 0.871395 | 4.75E-97 |
| RBM8A | 0.899453 | 3.3E-80 |
| SNX6 | 1.048117 | 1.37E-77 |
| PSIP1 | 0.736353 | 1.09E-76 |
| CCDC82 | 1.415735 | 9.22E-74 |
| MFAP1 | 0.886697 | 2.11E-69 |
| IK | 0.592052 | 9.57E-69 |
| PSMA4 | 0.603276 | 5.76E-67 |
| NUB1 | 0.921138 | 3.04E-64 |
| PHF14 | 0.760731 | 4.26E-64 |
| NIFK | 1.410417 | 6.26E-63 |
| RPL26 | 0.773784 | 1.49E-63 |
| PPIL4 | 0.914372 | 2.5E-59 |
| PHAX | 1.000856 | 1.67E-58 |
| LIMA1 | 0.676373 | 6.68E-59 |
| PPIG | 0.906238 | 1.48E-57 |
| SSB | 0.783715 | 1.61E-57 |
| HTATSF1 | 0.770073 | 2.61E-57 |
| POLR3GL | 0.870637 | 8.9E-57 |
| ZC3H13 | 0.75388 | 1.58E-55 |
| PTMA | 1.092841 | 5.94E-55 |
| AC013394.1 | 0.771411 | 8.16E-54 |
| MRFAP1L1 | 0.647219 | 1.76E-53 |
| UPF3B | 0.880165 | 1.68E-52 |
| PPP1R12A | 0.956314 | 3.47E-52 |
| MIER1 | 0.86231 | 4.06E-52 |
| FAM192A | 0.631382 | 4.46E-52 |
| BCLAF1 | 0.858497 | 9.81E-52 |
| SARNP | 0.803235 | 6.77E-51 |
| SSBP1 | 1.068655 | 6.34E-50 |
| ERLEC1 | 0.787283 | 3.58E-50 |
| CALD1 | 0.976209 | 4.19E-50 |
| LARP7 | 0.801446 | 4.1E-50 |
| ENAH | 1.379691 | 1.91E-49 |
| ANP32B | 0.799348 | 1.41E-49 |
| EIF3D | 0.600383 | 1.12E-49 |
| GALNT1 | 1.442204 | 1.4E-48 |
| NASP | 0.72095 | 4.05E-47 |
| KNOP1 | 1.098678 | 2.37E-46 |
| TCEAL4 | 0.809746 | 8.94E-46 |
| MMP14 | 1.757905 | 3.06E-45 |
| RBM7 | 0.910268 | 2.21E-45 |
| ESF1 | 0.955193 | 8.59E-45 |
| MORF4L1 | 0.785174 | 1.84E-44 |
| HSPB11 | 0.893081 | 3.56E-44 |
| IWS1 | 0.603841 | 1.91E-44 |
| FCF1 | 0.666168 | 7.03E-44 |
| RBM25 | 0.641297 | 2.31E-43 |
| PRPF38B | 0.796851 | 3.95E-43 |
| CTSO | 0.650567 | 2.89E-43 |
| LACTB | 0.862861 | 6.44E-43 |
| IQGAP1 | 1.105587 | 1.23E-42 |
| LUC7L3 | 0.808095 | 1.51E-42 |
| SHTN1 | 0.778506 | 1.1E-41 |
| MNS1 | 1.317729 | 4.5E-41 |
| AP3S1 | 0.969147 | 3.07E-41 |
| TOP2B | 0.61574 | 1.84E-41 |
| ZCCHC24 | 1.360891 | 6.92E-41 |
| ZRANB2 | 0.801398 | 8.14E-41 |
| RPL22P1 | 2.816271 | 8.75E-42 |
| UBLCP1 | 0.678603 | 1.23E-40 |
| SRI | 0.774962 | 1.42E-40 |
| MARCKS | 1.611426 | 3.97E-40 |
| NFYB | 0.749381 | 3.06E-40 |
| FAIM | 1.063418 | 8.68E-40 |
| FAM3C2 | 1.053061 | 1.6E-39 |
| PDCD10 | 0.815758 | 2.55E-39 |
| LEO1 | 0.806386 | 5.19E-39 |
| ZNF146 | 0.615769 | 3.36E-39 |
| CDH11 | 1.951861 | 1.75E-38 |
| CIR1 | 0.668559 | 5.18E-39 |
| RPL12 | 0.884018 | 5.97E-39 |
| KTN1 | 0.752569 | 1.49E-38 |
| PRKACB | 0.691713 | 3.73E-38 |
| CDC26 | 0.911191 | 6.87E-38 |
| ANKRD12 | 0.953789 | 8E-38 |
| ANTXR1 | 1.774414 | 1.75E-37 |
| CALU | 1.042365 | 1.27E-37 |
| EEF1A1P6 | 1.707706 | 8.36E-37 |
| CASP8 | 0.913456 | 2.76E-37 |
| UBE2Q2 | 0.834786 | 3.3E-37 |
| PLA2G4A | 1.598982 | 9.75E-37 |
| EEF1D | 1.713279 | 1E-36 |
| DDX50 | 0.691843 | 4.93E-37 |
| LACC1 | 1.078344 | 1.41E-36 |
| PIK3R3 | 0.754622 | 7.69E-37 |
| THAP12 | 0.736282 | 7.78E-37 |
| GALNT5 | 3.203417 | 7.8E-36 |
| VEZT | 0.633866 | 7.87E-37 |
| EIF5B | 0.711852 | 8.38E-37 |
| RAB23 | 0.977115 | 2.05E-36 |
| PIP4K2A | 1.180303 | 1.9E-36 |
| NARS | 0.799512 | 1.18E-36 |
| CCDC112 | 1.493769 | 5.49E-36 |
| NMI | 1.228902 | 5.04E-36 |
| KRCC1 | 0.616717 | 2.01E-36 |
| IFNAR2 | 1.726403 | 1.08E-35 |
| ZEB1 | 0.873695 | 4.59E-36 |
| RSRC2 | 0.593564 | 3.48E-36 |
| PPP1R3C | 1.022597 | 1.13E-35 |
| HNRNPD | 0.595581 | 4.81E-36 |
| RFX5 | 0.805226 | 7.84E-36 |
| TRPA1 | 2.368231 | 4.71E-35 |
| AC022149.1 | 2.888173 | 7.1E-37 |
| GPX8 | 1.772255 | 4.07E-35 |
| FGF7 | 2.307054 | 4.98E-35 |
| PFDN4 | 0.905262 | 2.82E-35 |
| STMN1 | 0.960653 | 2.7E-35 |
| USP1 | 0.696535 | 3.01E-35 |
| SMC6 | 0.662694 | 2.83E-35 |
| FSTL1 | 1.677746 | 7.65E-35 |
| EIF3A | 0.5996 | 3.59E-35 |
| ZNF260 | 0.754926 | 8.88E-35 |
| DCDC2 | 1.257774 | 1.48E-34 |
| CCPG1 | 0.630605 | 9.2E-35 |
| SSRP1 | 0.598757 | 2E-34 |
| PBDC1 | 0.696381 | 3.52E-34 |
| ANTXR2 | 1.238078 | 6.74E-34 |
| TXNDC9 | 0.806531 | 9E-34 |
| CALM2 | 0.706787 | 8.23E-34 |
| EEF1A1P5 | 1.130205 | 3.11E-33 |
| LTV1 | 0.669076 | 1.6E-33 |
| AC068631.2 | 2.73245 | 1.75E-32 |
| UTP14A | 0.669965 | 3.7E-33 |
| CCL11 | 2.705916 | 2.11E-32 |
| ITGB1 | 0.966826 | 4.31E-33 |
| CPA3 | 3.400498 | 8.39E-33 |
| ZNF14 | 0.654209 | 6.69E-33 |
| DEK | 0.838475 | 8.01E-33 |
| SESN3 | 0.738947 | 8.01E-33 |
| CADM3 | 3.306411 | 2.07E-32 |
| CCND2 | 2.096912 | 4.49E-32 |
| KIAA1551 | 1.184554 | 3.79E-32 |
| PLEKHA4 | 1.497795 | 6.57E-32 |
| TCEAL9 | 0.777462 | 4.01E-32 |
| NAP1L1 | 0.710822 | 6.4E-32 |
| ZKSCAN4 | 0.702109 | 9.78E-32 |
| COL10A1 | 3.45592 | 1.85E-31 |
| SMIM10 | 1.463322 | 3.18E-31 |
| DNAJC10 | 0.664564 | 1.13E-31 |
| UACA | 0.695345 | 1.35E-31 |
| SMC3 | 0.590101 | 1.43E-31 |
| HDGFL3 | 0.839024 | 2.39E-31 |
| PROS1 | 0.728227 | 1.9E-31 |
| MS4A2 | 2.39133 | 1.21E-30 |
| SMARCA5 | 0.712587 | 2.67E-31 |
| SLFN5 | 1.463529 | 7.02E-31 |
| FOS | -5.50799 | 1.4E-178 |
| AL162151.2 | -9.07986 | 2.9E-157 |
| ATP5F1E | -1.56743 | 1.7E-156 |
| NR4A1 | -4.28653 | 8.7E-133 |
| NDUFAF3 | -1.69461 | 5.5E-130 |
| POLR2L | -1.54938 | 3.5E-109 |
| CHCHD2 | -1.00429 | 6.3E-102 |
| MTATP6P1 | -2.75924 | 4.06E-92 |
| RPS29 | -1.61847 | 2.45E-92 |
| TMEM256 | -1.90232 | 1.55E-87 |
| TOMM7 | -1.53274 | 1.86E-85 |
| COX5B | -1.35457 | 2.75E-79 |
| SAP18 | -1.15128 | 2.3E-79 |
| ERRFI1 | -3.20226 | 9.18E-71 |
| ACTR1B | -0.64832 | 3.71E-74 |
| ATP6V0B | -0.94391 | 9.37E-72 |
| EGR1 | -3.69657 | 1.29E-70 |
| RPLP1 | -1.43427 | 3.45E-71 |
| PIM3 | -1.42825 | 1.7E-70 |
| MST1 | -1.66968 | 1.24E-69 |
| CDIPT | -0.70444 | 2.48E-70 |
| CISH | -2.02699 | 4.55E-69 |
| ESRRA | -1.15799 | 4.3E-69 |
| DVL1 | -0.74451 | 2.24E-68 |
| NDUFB11 | -0.92651 | 4.62E-66 |
| C4orf3 | -0.8619 | 1.43E-65 |
| JAGN1 | -0.92503 | 1.57E-64 |
| RBKS | -1.80785 | 6.63E-64 |
| ZFP36 | -2.53175 | 1.29E-63 |
| MDH2 | -1.0769 | 4.8E-64 |
| EIF1 | -0.59349 | 4.84E-64 |
| NR4A2 | -3.35316 | 1.33E-61 |
| OPLAH | -1.35521 | 8.09E-62 |
| UBE2K | -0.8707 | 7.11E-61 |
| RPL36 | -1.06945 | 5.72E-60 |
| METRN | -1.78386 | 2.5E-59 |
| WDR13 | -0.86168 | 3.92E-59 |
| KLHL21 | -1.15746 | 9.67E-59 |
| ATP6AP1 | -0.79352 | 1.55E-58 |
| COX7C | -1.0145 | 2.84E-58 |
| GRINA | -0.95816 | 2.62E-58 |
| GCGR | -2.20891 | 2.84E-57 |
| NDUFB6 | -1.34607 | 2.72E-57 |
| SLC25A6 | -0.76529 | 1.6E-57 |
| G6PC | -3.29606 | 7.28E-56 |
| MFSD3 | -1.51091 | 6.66E-56 |
| NUCB1 | -0.91365 | 1.84E-56 |
| NDUFS8 | -0.94829 | 7.71E-56 |
| MRPS24 | -0.98372 | 1.37E-55 |
| PQLC1 | -1.15277 | 1.47E-54 |
| COQ4 | -0.82729 | 2E-54 |
| RPS28P7 | -2.40132 | 3.75E-53 |
| NAA38 | -0.97455 | 9.53E-54 |
| TECR | -1.02482 | 1.03E-53 |
| FOSB | -4.35247 | 9.23E-53 |
| EMC10 | -0.69178 | 1.39E-53 |
| WDTC1 | -0.60941 | 1.76E-53 |
| TMEM258 | -0.96308 | 7.16E-53 |
| LRRC75A | -0.98676 | 2.48E-52 |
| NR0B2 | -2.98451 | 5.21E-51 |
| CYR61 | -2.60287 | 2.4E-51 |
| NDUFC1 | -1.09073 | 1.06E-51 |
| RETREG2 | -0.61826 | 6.6E-52 |
| RORC | -1.64577 | 9.04E-51 |
| RPS28 | -0.94138 | 7.25E-51 |
| DENND1C | -0.86482 | 1.13E-50 |
| STARD8 | -0.99105 | 2.4E-50 |
| ZBTB45P1 | -1.47918 | 1.92E-49 |
| ELOB | -0.89507 | 4.6E-50 |
| ID2 | -1.15328 | 1.17E-49 |
| AP3D1 | -0.66171 | 5.22E-50 |
| ALDOA | -0.66275 | 8.71E-50 |
| ABHD14B | -0.97186 | 1.36E-48 |
| ISOC2 | -1.29271 | 2.93E-48 |
| TMEM129 | -0.77373 | 3.98E-48 |
| CISD3 | -1.26926 | 1.12E-47 |
| ALKBH7 | -0.95204 | 9.45E-48 |
| MFSD4A | -1.73091 | 1.98E-47 |
| PMM1 | -0.90044 | 8.33E-48 |
| GGACT | -2.43571 | 4.55E-47 |
| CSRNP1 | -1.59592 | 3.92E-47 |
| GUCD1 | -0.8461 | 1.7E-47 |
| HSPBP1 | -0.77408 | 3.92E-47 |
| DNAJC15 | -1.11332 | 8.6E-47 |
| ATP5MC2 | -0.95589 | 1.02E-46 |
| DNASE1 | -1.7242 | 4.08E-46 |
| ATP5MC3 | -0.97935 | 2.37E-46 |
| NDUFS7 | -1.01018 | 3.56E-46 |
| NUDT22 | -0.87073 | 4E-46 |
| CYFIP2 | -1.44154 | 7.34E-46 |
| SIK1B | -2.37067 | 2.43E-45 |
| NME3 | -0.99676 | 7.91E-46 |
| PTGER3 | -1.60442 | 1.43E-45 |
| DIAPH1 | -0.61664 | 5.16E-46 |
| VAMP2 | -0.61062 | 7.49E-46 |
| UQCC3 | -0.96998 | 2.66E-45 |
| C2orf68 | -0.8847 | 2.69E-45 |
| COX7A2 | -0.86723 | 2.43E-45 |
| MRPL53 | -0.99586 | 8.16E-45 |
| DOHH | -0.86354 | 9.56E-45 |
| COPE | -0.59218 | 4.7E-45 |
| LZTS3 | -1.05049 | 6.88E-44 |
| WDR34 | -0.69704 | 5.37E-44 |
| AP5B1 | -0.70525 | 6.41E-44 |
| ATF3 | -2.99435 | 1.31E-44 |
| TPST2 | -0.86736 | 1.74E-43 |
| MCRIP2 | -1.25663 | 2.86E-43 |
| MRPL4 | -0.6824 | 1.22E-43 |
| MMP24OS | -0.94581 | 2.46E-43 |
| RFNG | -0.7534 | 1.85E-43 |
| MRPS6 | -0.97617 | 2.84E-43 |
| HPN | -1.17457 | 3.11E-43 |
| PLA2G15 | -0.82042 | 3.05E-43 |
| IP6K3 | -2.58799 | 3.45E-42 |
| SLC23A3 | -2.45696 | 2.08E-42 |
| UBALD1 | -0.94231 | 7.4E-43 |
| COMMD6 | -0.99249 | 8.76E-43 |
| UBB | -0.80158 | 5.79E-43 |
| C19orf70 | -0.79747 | 1.8E-42 |
| MRPL55 | -0.8379 | 2.82E-42 |
| HERPUD1 | -0.78747 | 1.84E-42 |
| UQCR11 | -0.88072 | 3.99E-42 |
| LSM4 | -0.74367 | 3.36E-42 |
| TMEM179B | -0.61773 | 2.82E-42 |
| GALT | -0.78973 | 4.36E-42 |
| TMEM219 | -0.8425 | 4.54E-42 |
| WBP2 | -0.60349 | 2.8E-42 |
| D2HGDH | -1.26503 | 8.16E-42 |
| AC005726.1 | -1.7877 | 1.69E-41 |
| TRUB2 | -0.88385 | 7.42E-42 |
| TRIM28 | -0.71527 | 4.8E-42 |
| FUZ | -0.97783 | 1.12E-41 |
| MRPS12 | -0.87797 | 1.45E-41 |
| TMEM82 | -1.56191 | 3.99E-41 |
| GPS1 | -0.83814 | 1.2E-41 |
| GALK1 | -1.31533 | 2.95E-41 |
| AC068946.1 | -2.06725 | 9.63E-41 |
| FASTK | -0.7323 | 1.7E-41 |
| BRAT1 | -0.65806 | 1.9E-41 |
| STK16 | -0.86352 | 3.05E-41 |
| RNF123 | -0.81046 | 3.4E-41 |
| SNX27 | -0.77661 | 4.43E-41 |
| GDF15 | -2.2679 | 2.23E-40 |
| HAAO | -1.41814 | 1.36E-40 |
| NAGLU | -0.92802 | 8.44E-41 |
| TMEM161A | -0.89647 | 1.09E-40 |
| BTG2 | -1.90218 | 2.46E-40 |
| NDUFB4 | -0.79689 | 9.67E-41 |
| THEM6 | -1.14088 | 1.82E-40 |
| BLCAP | -0.60873 | 7.25E-41 |
