# Supplementary material for: Inhibition of HDAC6 With CAY10603 Ameliorates Diabetic Kidney Disease by Suppressing NLRP3 Inflammasome
Source: Front Pharmacol. 2022 Jul 14;13:938391. doi: 10.3389/fphar.2022.938391 (PMC9332914; doi:10.3389/fphar.2022.938391)
Supplement: Supplementary file 6 [file DataSheet1.PDF]

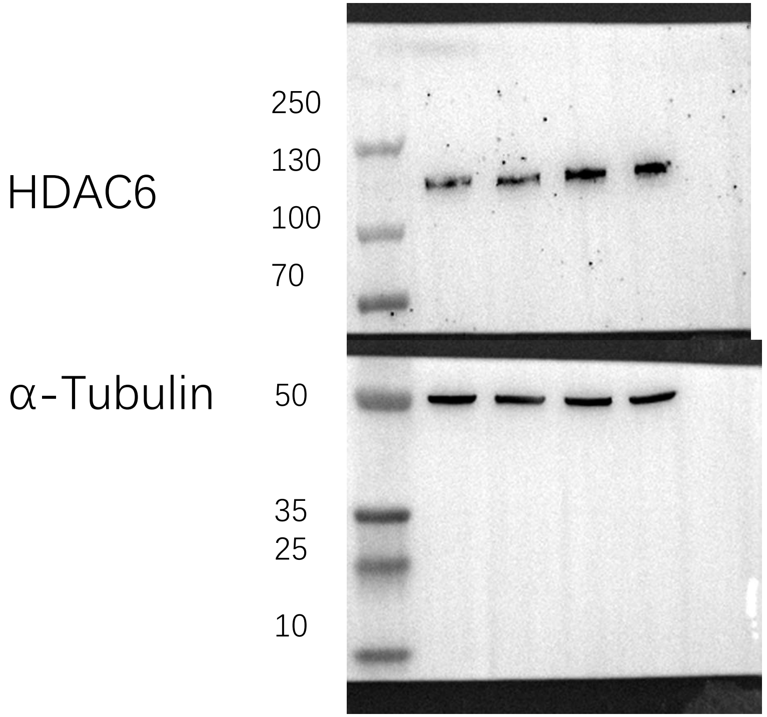
HDAC6
α-Tubulin
250
130
100
70
50
35
25
10

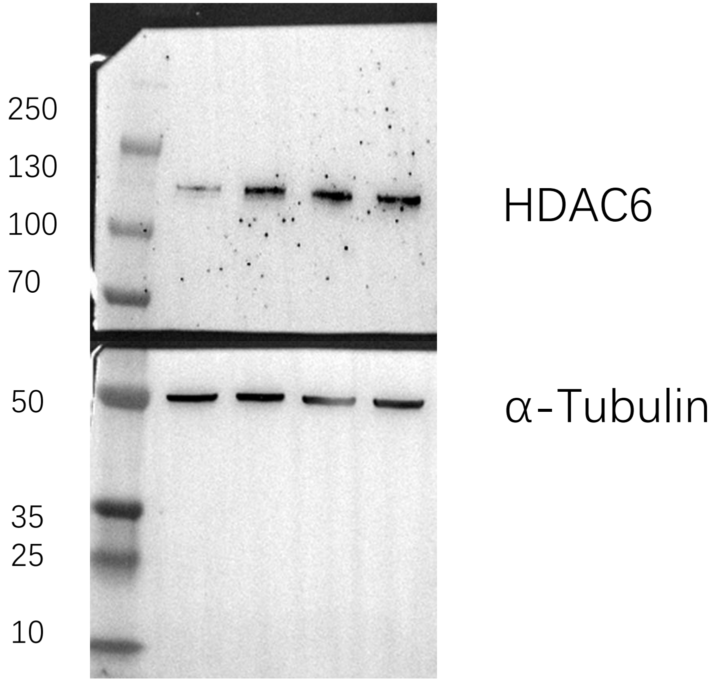
250
130
100
70
HDAC6
50
α-Tubulin
35
25
10

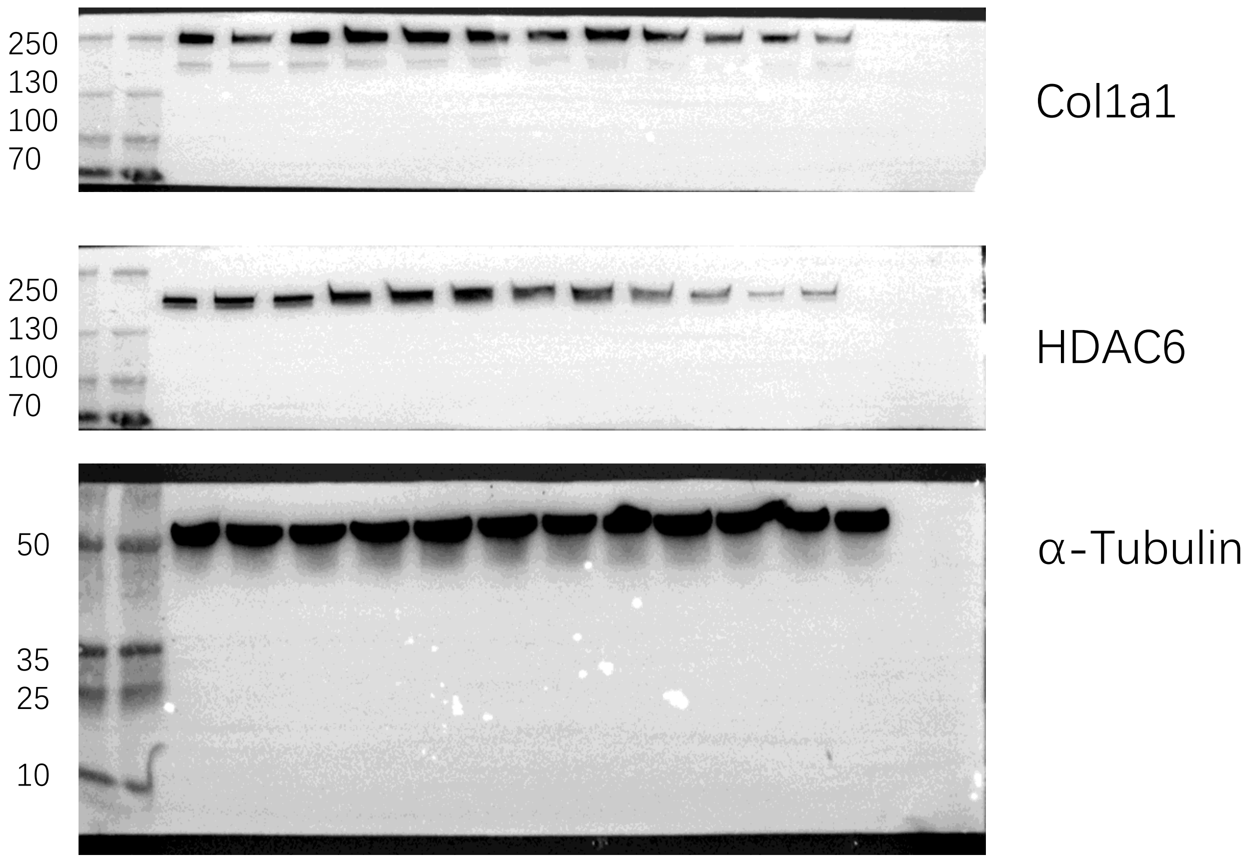
250
130
100
70
Col1a1
250
130
100
70
HDAC6
50
35
25
10
α-Tubulin

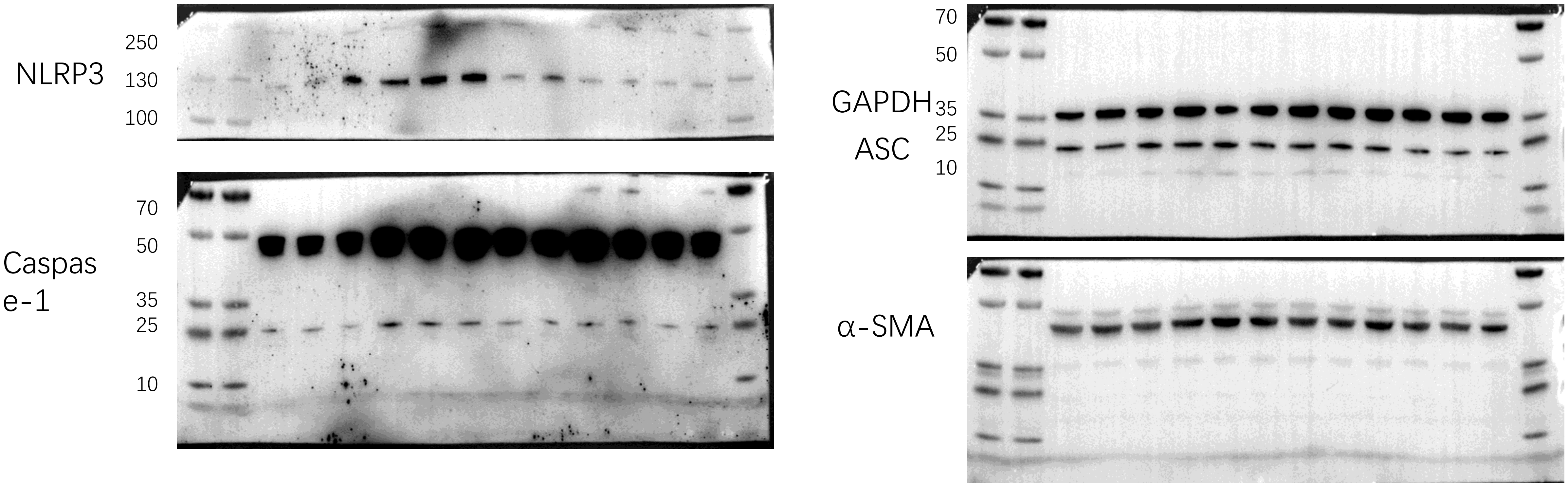
NLRP3
250
130
100
Caspas
e-1
70
50
35
25
10
GAPDH
ASC
70
50
35
25
10
α-SMA

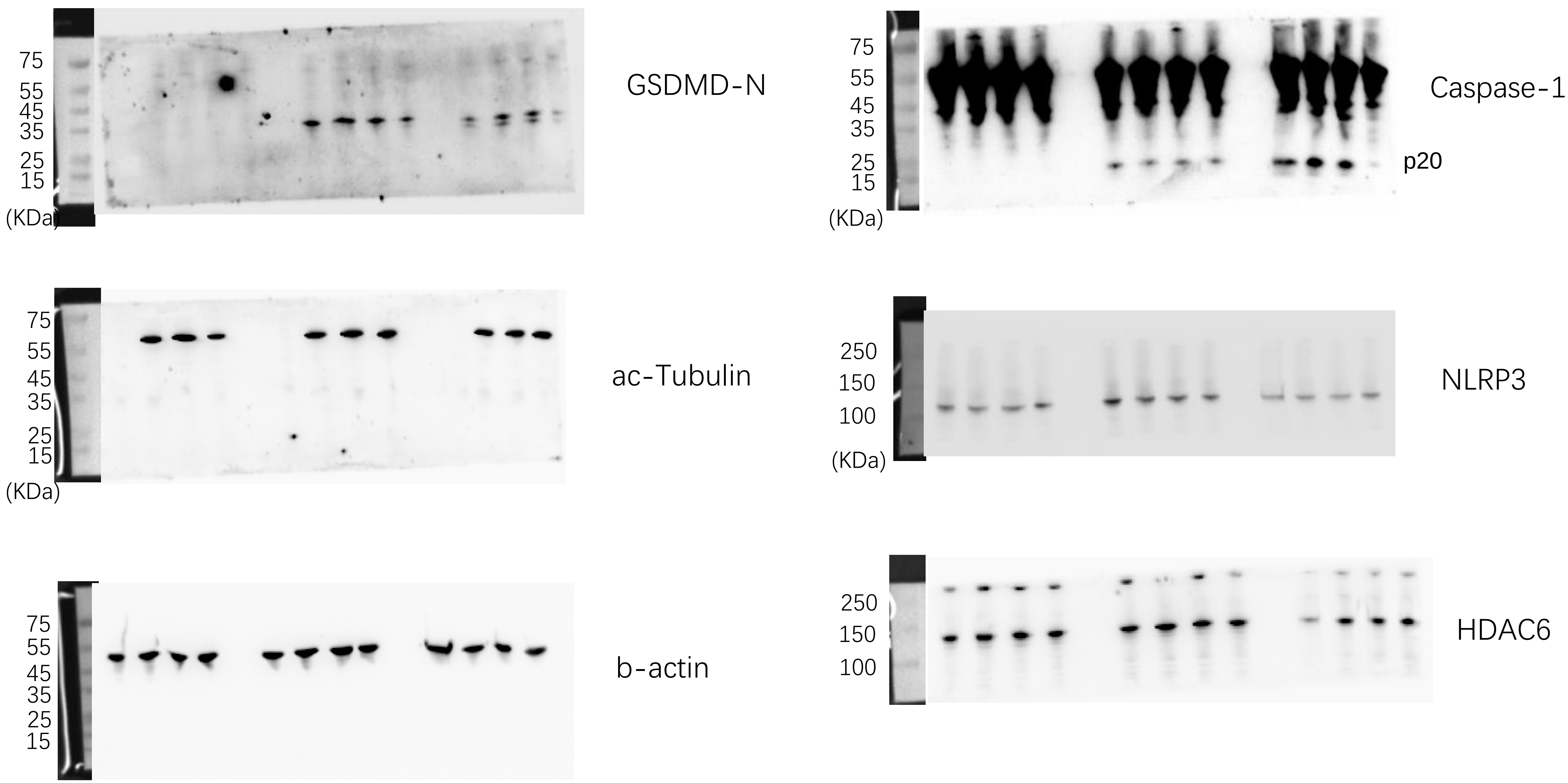
75
55
45
35
25
15
(KDa)
GSDMD-N
75
55
45
35
25
15
(KDa)
Caspase-1
p20
75
55
45
35
25
15
(KDa)
ac-Tubulin
250
150
100
(KDa)
NLRP3
75
55
45
35
25
15
b-actin
250
150
100
HDAC6
