# Supplementary material for: Inhibition of HDAC6 With CAY10603 Ameliorates Diabetic Kidney Disease by Suppressing NLRP3 Inflammasome
Source: Front Pharmacol. 2022 Jul 14;13:938391. doi: 10.3389/fphar.2022.938391 (PMC9332914; doi:10.3389/fphar.2022.938391)
Supplement: Supplementary file 7 [file Table3.DOCX]

**Supplementary table 3：The top 150 upregulated genes and top 150 down-regulated genes in early stage DN compared with healthy controls (fold change>1.5, p value <0.05).**

| gene_id | Fold Change(log2) | padj |
| --- | --- | --- |
| RPS24 | 1.231474 | 2.5E-168 |
| AC116533.1 | 7.314526 | 7.2E-126 |
| SET | 0.944537 | 7.4E-118 |
| PHAX | 1.149938 | 8.44E-91 |
| SSBP1 | 1.316208 | 8.9E-89 |
| MFAP1 | 0.914515 | 1.05E-86 |
| RBM8A | 0.85935 | 1.14E-86 |
| LACTB | 1.107086 | 1.04E-82 |
| PSMA4 | 0.619714 | 3.12E-83 |
| MRFAP1L1 | 0.743705 | 5.37E-83 |
| SMARCE1 | 0.740914 | 6.5E-83 |
| PPIL4 | 0.965921 | 7E-78 |
| SSB | 0.834517 | 6.67E-77 |
| HTATSF1 | 0.813774 | 1.92E-75 |
| PPIG | 0.942587 | 1.55E-73 |
| NIFK | 1.394089 | 2E-72 |
| NARS | 1.042541 | 9.53E-73 |
| CCDC82 | 1.282465 | 1.18E-71 |
| CDC26 | 1.157091 | 3.56E-71 |
| PNRC2 | 0.624942 | 4.35E-71 |
| RPL26 | 0.748728 | 1.51E-70 |
| SNX6 | 0.915771 | 3E-70 |
| PSIP1 | 0.648601 | 2.55E-70 |
| ESF1 | 1.091649 | 1.98E-68 |
| MORF4L1 | 0.897125 | 2.38E-68 |
| CIR1 | 0.817784 | 4.56E-68 |
| RSL1D1 | 0.677219 | 1.56E-66 |
| AC013394.1 | 0.784716 | 3.92E-65 |
| LARP7 | 0.841613 | 4.23E-65 |
| UPF3B | 0.900366 | 3.49E-64 |
| FAM192A | 0.639434 | 5.98E-63 |
| LEO1 | 0.937249 | 1.82E-61 |
| SARNP | 0.814268 | 1.77E-61 |
| PSMC1 | 0.914139 | 4.47E-60 |
| HSPB11 | 0.95083 | 1.63E-58 |
| TCEAL4 | 0.843628 | 1.62E-58 |
| EIF5B | 0.82553 | 6.82E-58 |
| LUC7L3 | 0.867251 | 1.09E-57 |
| ANP32B | 0.7922 | 1.83E-57 |
| THOC2 | 0.637002 | 1.4E-57 |
| PSMC6 | 0.627417 | 4.43E-56 |
| RPL22P1 | 3.066409 | 5.31E-57 |
| IWS1 | 0.623312 | 1.45E-55 |
| KTN1 | 0.829469 | 5.67E-55 |
| PSMC3 | 0.912129 | 1.07E-54 |
| PRPF18 | 0.83791 | 3.57E-54 |
| DPM1 | 0.699356 | 2.55E-54 |
| PHF14 | 0.643961 | 6.15E-54 |
| NUB1 | 0.773801 | 8.14E-54 |
| ZC3H13 | 0.682681 | 7.41E-54 |
| PDCD10 | 0.878504 | 2.1E-53 |
| HMGN5 | 1.25342 | 1.74E-51 |
| SEC62 | 0.946932 | 7.58E-52 |
| POLR3GL | 0.763997 | 2.96E-51 |
| PAIP1 | 0.651714 | 1.83E-51 |
| EEF1A1P6 | 1.871286 | 5.09E-50 |
| KRCC1 | 0.674621 | 8.19E-51 |
| NSRP1 | 0.691098 | 6.91E-50 |
| EIF3A | 0.657026 | 1.67E-49 |
| MT-ND6 | 1.756504 | 7.71E-48 |
| PBDC1 | 0.765075 | 6.42E-48 |
| AIMP1 | 0.729913 | 1.36E-47 |
| TCEAL8 | 0.675191 | 1.25E-47 |
| NSA2 | 0.700287 | 1.66E-47 |
| AP3S1 | 0.954209 | 4.9E-47 |
| HAT1 | 0.617125 | 3.63E-47 |
| FAM3C2 | 1.049229 | 5.93E-46 |
| EEF1D | 1.76229 | 2.17E-45 |
| LTV1 | 0.725652 | 9.6E-46 |
| KNOP1 | 1.000339 | 2.76E-45 |
| FCF1 | 0.625807 | 1.16E-45 |
| HNRNPD | 0.618653 | 1.12E-45 |
| PIK3R3 | 0.772861 | 2.76E-45 |
| UBLCP1 | 0.662625 | 2.76E-45 |
| RBM7 | 0.836004 | 4.4E-45 |
| SNX14 | 0.713579 | 3.29E-45 |
| CWC22 | 0.650162 | 6.57E-45 |
| MTCO1P12 | 3.885303 | 4.43E-44 |
| TOP2B | 0.590196 | 5.67E-45 |
| TXNDC9 | 0.857325 | 1.84E-44 |
| NFYB | 0.727654 | 1.34E-44 |
| DDX50 | 0.701233 | 1.67E-44 |
| UTP3 | 0.629784 | 1.53E-44 |
| THAP12 | 0.743524 | 4.44E-44 |
| CCPG1 | 0.655279 | 4.65E-44 |
| ZNF14 | 0.702993 | 1.17E-43 |
| SRI | 0.740564 | 1.13E-43 |
| CEBPZ | 0.861936 | 1.45E-43 |
| RPL6 | 0.599 | 1.07E-43 |
| RBM34 | 0.868019 | 2.59E-43 |
| PTMA | 0.887879 | 2.92E-43 |
| MPHOSPH10 | 0.684103 | 4.01E-43 |
| PPID | 0.940461 | 9.85E-43 |
| ARL1 | 0.769139 | 1.21E-42 |
| ERLEC1 | 0.66668 | 1.11E-42 |
| RPF1 | 0.599821 | 1.2E-42 |
| BZW1P2 | 0.932283 | 3.62E-42 |
| RAB10 | 0.751779 | 3.68E-42 |
| RSRC2 | 0.591561 | 3E-42 |
| C11orf98 | 0.709629 | 1.75E-41 |
| RNF13 | 0.734418 | 2.62E-41 |
| POLR2K | 0.644018 | 7.41E-41 |
| SF3B6 | 0.616697 | 8.36E-41 |
| LLPH | 0.748721 | 1.37E-40 |
| EIF4E | 0.59341 | 1.7E-40 |
| FKBP3 | 0.627064 | 2.85E-40 |
| AC068631.2 | 2.800179 | 5.98E-39 |
| TOPORS | 0.651142 | 3.9E-40 |
| DNTTIP2 | 0.720383 | 1.34E-39 |
| BCLAF1 | 0.688293 | 1.21E-39 |
| CALD1 | 0.791911 | 2.52E-39 |
| EIF2S2 | 0.604558 | 3.61E-39 |
| SHTN1 | 0.689478 | 1.1E-38 |
| PPP1R12A | 0.753638 | 1.25E-38 |
| HPF1 | 0.922276 | 5.11E-38 |
| MIER1 | 0.678326 | 2.61E-38 |
| RPL12 | 0.805308 | 2.84E-38 |
| FYN | 0.706015 | 5.81E-38 |
| UTP14A | 0.663221 | 9.45E-38 |
| ACTR10 | 0.622236 | 7.76E-38 |
| EEF1A1P5 | 1.111128 | 4.11E-37 |
| WBP4 | 0.639719 | 2.42E-37 |
| TTC4 | 0.892649 | 8.08E-37 |
| ANKRD12 | 0.860834 | 2.03E-36 |
| DNAJC21 | 0.656211 | 2.12E-36 |
| ERBIN | 0.745469 | 2.9E-36 |
| CUL2 | 0.616893 | 3.63E-36 |
| ZNF260 | 0.709726 | 4.71E-36 |
| FRG1 | 0.648512 | 4.83E-36 |
| TRMT10C | 0.925432 | 8.59E-36 |
| UTP11 | 0.802135 | 8.01E-36 |
| SMC6 | 0.614079 | 1.36E-35 |
| PROS1 | 0.713642 | 1.71E-35 |
| UBE2V2 | 0.735779 | 2.35E-35 |
| SRP72 | 0.718543 | 3.41E-35 |
| PPP1R3C | 0.932949 | 7.94E-35 |
| CALM2 | 0.662992 | 4.33E-35 |
| XRCC4 | 0.810191 | 9.08E-35 |
| RPS23 | 0.721103 | 5.99E-35 |
| CEP63 | 0.642267 | 1.05E-34 |
| ZC3H7A | 0.695442 | 1.22E-34 |
| SENP2 | 0.873021 | 2.99E-34 |
| TMEM126B | 0.617059 | 2.37E-34 |
| CWF19L2 | 0.743055 | 5.04E-34 |
| ZBTB14 | 0.699347 | 5.31E-34 |
| PFDN4 | 0.819664 | 9.66E-34 |
| TCEAL9 | 0.733949 | 1.2E-33 |
| NDUFAF8 | 0.858477 | 1.88E-33 |
| GALNT1 | 1.09251 | 2.66E-33 |
| PIP4K2A | 1.036496 | 3.02E-33 |
| FOS | -5.95024 | 2.6E-243 |
| AL162151.2 | -9.28339 | 6E-211 |
| NR4A1 | -4.36645 | 1.2E-161 |
| ATP5F1E | -1.31734 | 6.1E-131 |
| EGR1 | -4.24641 | 4.1E-108 |
| ZFP36 | -2.95172 | 4E-101 |
| NDUFAF3 | -1.36205 | 1.9E-99 |
| RPLP1 | -1.53583 | 1.44E-96 |
| POLR2L | -1.32005 | 5.45E-94 |
| ERRFI1 | -3.20767 | 3.12E-83 |
| RPS29 | -1.43931 | 1.19E-86 |
| NR4A2 | -3.65724 | 6.44E-85 |
| CYR61 | -3.00614 | 1.09E-79 |
| CHCHD2 | -0.81601 | 6.16E-80 |
| PIM3 | -1.38946 | 4.48E-79 |
| ULK1 | -1.17317 | 3.12E-79 |
| DVL1 | -0.73261 | 2.19E-78 |
| RPL36 | -1.09944 | 5.26E-75 |
| ACTR1B | -0.59798 | 7.03E-75 |
| CDIPT | -0.66442 | 3.43E-74 |
| BTG2 | -2.34819 | 2.2E-71 |
| UBE2K | -0.8708 | 4.79E-72 |
| FOSB | -4.64132 | 3.52E-68 |
| KLHL21 | -1.12607 | 6.96E-66 |
| MTATP6P1 | -2.13037 | 3.29E-65 |
| EMC10 | -0.70512 | 6.97E-66 |
| JUN | -2.30681 | 5.1E-64 |
| JUNB | -2.49398 | 2.44E-62 |
| TMEM256 | -1.46729 | 2.07E-62 |
| TOMM7 | -1.19849 | 3.22E-62 |
| RHOB | -1.26113 | 3.7E-62 |
| TMEM258 | -0.93948 | 1.45E-59 |
| TRIM28 | -0.78726 | 6.67E-60 |
| CISH | -1.71022 | 2.09E-58 |
| SIK1B | -2.48119 | 5.06E-58 |
| RPS28P7 | -2.30029 | 1.4E-57 |
| ATF3 | -3.20952 | 2.93E-59 |
| TMEM259 | -0.61432 | 4.49E-58 |
| JUND | -1.31847 | 9.46E-56 |
| PER1 | -2.08512 | 1.22E-54 |
| MAFK | -1.10127 | 5.99E-55 |
| CSRNP1 | -1.58088 | 1.55E-54 |
| LRRC75A | -0.92499 | 1.67E-54 |
| FBRSL1 | -1.10846 | 3.41E-54 |
| SLC27A1 | -0.65777 | 1.68E-54 |
| BRAT1 | -0.69178 | 7.55E-54 |
| RPS28 | -0.88127 | 7.91E-53 |
| NME3 | -0.98034 | 2.25E-52 |
| WDR13 | -0.74474 | 1.39E-52 |
| SQSTM1 | -0.78345 | 1.6E-52 |
| IER2 | -1.53226 | 2.04E-51 |
| IRS2 | -1.09463 | 1.82E-51 |
| SEC14L1 | -0.68759 | 1.4E-51 |
| COL6A1 | -0.7288 | 2.96E-51 |
| METRN | -1.50827 | 1.98E-50 |
| OPLAH | -1.12327 | 1.35E-50 |
| RHPN1 | -0.88413 | 1.89E-50 |
| ATP6V0B | -0.72375 | 1.98E-50 |
| FAM189B | -0.62389 | 2.91E-50 |
| MAP1LC3A | -0.94318 | 1.04E-49 |
| GADD45B | -1.90284 | 3.58E-49 |
| GDF15 | -2.31136 | 5.66E-49 |
| FOXK1 | -0.63668 | 1.04E-49 |
| C2orf68 | -0.84904 | 2.94E-49 |
| AP5B1 | -0.68564 | 5.19E-49 |
| ATP6AP1 | -0.66526 | 5.19E-49 |
| NAA38 | -0.84735 | 2.18E-48 |
| COQ4 | -0.71346 | 3.76E-48 |
| PQLC1 | -0.99011 | 6.98E-48 |
| SPNS1 | -0.69387 | 4.64E-48 |
| EFNA1 | -1.26165 | 2.37E-47 |
| SLC39A13 | -0.6919 | 1.36E-47 |
| INTS1 | -0.88453 | 1.97E-47 |
| HES1 | -1.44724 | 7.05E-47 |
| TMEM8B | -0.81616 | 9.79E-47 |
| RASD1 | -2.80975 | 5.87E-46 |
| ISYNA1 | -1.05553 | 4.83E-46 |
| SUMO2 | -0.62416 | 2.94E-46 |
| CEBPD | -1.47404 | 8.12E-45 |
| COMMD6 | -0.93349 | 9.46E-45 |
| C4orf3 | -0.6529 | 6E-45 |
| TMEM129 | -0.6851 | 7.55E-45 |
| WASH3P | -1.03204 | 4.25E-44 |
| KIFC2 | -0.97717 | 9.78E-44 |
| RAB43 | -1.11649 | 1.8E-43 |
| TMEM161A | -0.85241 | 1.68E-43 |
| MDH2 | -0.80995 | 2.35E-43 |
| ABTB1 | -0.8877 | 3.46E-43 |
| JAGN1 | -0.68906 | 5.56E-43 |
| ZBTB45P1 | -1.25275 | 2.07E-42 |
| HSPBP1 | -0.67667 | 7.98E-43 |
| NUDT22 | -0.76583 | 1.85E-42 |
| PPP1R12C | -0.60396 | 3.34E-42 |
| DNAJC15 | -0.95899 | 2.87E-41 |
| LRP10 | -0.59492 | 2.2E-41 |
| TSC22D3 | -1.79792 | 1.42E-40 |
| SAP18 | -0.75559 | 4.55E-41 |
| FASTK | -0.66931 | 4.43E-41 |
| WDR34 | -0.61354 | 1.67E-40 |
| EDC4 | -0.74094 | 2.85E-40 |
| TMEM219 | -0.75265 | 7.31E-40 |
| DOHH | -0.73785 | 6.9E-39 |
| COX5B | -0.86136 | 1.3E-38 |
| DGAT1 | -0.64588 | 2.07E-38 |
| FZR1 | -0.66887 | 2.87E-38 |
| MST1 | -1.1201 | 9.81E-38 |
| HDAC5 | -0.67161 | 5.46E-38 |
| RBKS | -1.2624 | 1.64E-37 |
| DDIT4 | -1.50469 | 2.9E-37 |
| AC007318.2 | -1.91491 | 8.05E-37 |
| SPSB3 | -0.72912 | 4.52E-37 |
| AC018638.5 | -1.35789 | 2.09E-36 |
| GCGR | -1.60392 | 2.84E-36 |
| MFSD3 | -1.11225 | 2.26E-36 |
| NUCB1 | -0.6713 | 1.62E-36 |
| SREBF2 | -0.6337 | 3.89E-36 |
| NDUFB11 | -0.62381 | 4.53E-36 |
| HERPUD1 | -0.66022 | 1.51E-35 |
| SERINC2 | -0.7361 | 1.8E-35 |
| ESRRA | -0.75042 | 5.58E-35 |
| RGS2 | -1.64041 | 2.37E-34 |
| PDK4 | -2.61754 | 2.07E-34 |
| PTP4A1 | -0.93403 | 2.22E-34 |
| AC018638.4 | -1.07109 | 4.25E-34 |
| AP5Z1 | -0.72647 | 1.89E-34 |
| TPST2 | -0.70706 | 1.77E-34 |
| LMNA | -0.72737 | 2.11E-34 |
| BCAM | -0.76587 | 3.29E-34 |
| DCPS | -0.7912 | 5.7E-34 |
| EPOR | -0.9359 | 8.72E-34 |
| GRINA | -0.66794 | 4.77E-34 |
| FOSL2 | -1.29194 | 1.08E-33 |
| ZDHHC24 | -0.74863 | 9.41E-34 |
| WASH9P | -1.32977 | 2.68E-33 |
| POLRMT | -0.8628 | 1.06E-33 |
| PIGO | -0.65989 | 9.91E-34 |
| ENKD1 | -0.72171 | 1.85E-33 |
| MRPS24 | -0.6952 | 1.99E-33 |
| KLF6 | -1.52199 | 1.24E-32 |
| ELOB | -0.66313 | 5.8E-33 |
| LMF2 | -0.66416 | 5.83E-33 |
| TSPAN14 | -0.59663 | 7.2E-33 |
| CHPF | -0.74423 | 1.01E-32 |
| SPPL2B | -0.68711 | 2.67E-32 |
| NR0B2 | -2.12764 | 1.76E-31 |
| MFSD10 | -1.04064 | 5.94E-32 |
| ID2 | -0.84462 | 5.82E-32 |
| SLC52A2 | -0.71348 | 8.42E-32 |
| NDUFS8 | -0.65318 | 6.66E-32 |
| MZT2B | -0.67391 | 9.68E-32 |
