# Supplementary material for: Inhibition of HDAC6 With CAY10603 Ameliorates Diabetic Kidney Disease by Suppressing NLRP3 Inflammasome
Source: Front Pharmacol. 2022 Jul 14;13:938391. doi: 10.3389/fphar.2022.938391 (PMC9332914; doi:10.3389/fphar.2022.938391)
Supplement: Supplementary file 8 [file Table4.DOCX]

Supplementary table：The list of top 100 small molecule compound with highest negative score predicted by using regulated genes between early stage DN and healthy controls.

| Score | Type | ID | Name | Description |
| --- | --- | --- | --- | --- |
| -98.8 | cp | BRD-K15108141 | gemcitabine | Ribonucleotide reductase inhibitor |
| -98.1 | cp | BRD-K08554278 | bisbenzimide | DNA binding agent |
| -98.06 | cp | BRD-U82589721 | HG-5-113-01 | Protein kinase inhibitor |
| -97.82 | cp | BRD-A71459254 | cymarin | ATPase inhibitor |
| -97.78 | cp | BRD-A30437061 | camptothecin | Topoisomerase inhibitor |
| -97.77 | cp | BRD-K02130563 | panobinostat | HDAC inhibitor |
| -97.7 | cp | BRD-K06426971 | ryuvidine | Histone lysine methyltransferase inhibitor |
| -97.62 | cp | BRD-K92991072 | PAC-1 | Caspase activator |
| -97.57 | cp | BRD-A59985574 | topotecan | Topoisomerase inhibitor |
| -97.5 | cp | BRD-A79465854 | auranofin | NFkB pathway inhibitor |
| -97.43 | cp | BRD-K63606607 | bufalin | ATPase inhibitor |
| -97.36 | cp | BRD-A93236127 | digitoxin | ATPase inhibitor |
| -97.32 | cp | BRD-A45333398 | periplocymarin | Apoptosis stimulant |
| -97.25 | cp | BRD-K18518344 | digitoxigenin | ATPase inhibitor |
| -97.15 | cp | BRD-K60623809 | SU-11652 | Tyrosine kinase inhibitor |
| -96.86 | cp | BRD-K64606589 | apicidin | HDAC inhibitor |
| -96.83 | cp | BRD-A02481876 | importazole | Importin-beta transport receptor inhibitor |
| -96.76 | cp | BRD-A94756469 | digoxin | ATPase inhibitor |
| -96.69 | cp | BRD-K15409150 | penfluridol | T-type calcium channel blocker |
| -96.48 | cp | BRD-K04853698 | LDN-193189 | Serine/threonine kinase inhibitor |
| -96.44 | cp | BRD-K17140735 | SCH-79797 | Proteasome inhibitor |
| -96.3 | cp | BRD-K69932463 | AZD-8055 | MTOR inhibitor |
| -96.21 | cp | BRD-A80502530 | cinobufagin | ATPase inhibitor |
| -96.16 | cp | BRD-A34806832 | proscillaridin | ATPase inhibitor |
| -96.16 | cp | BRD-A68930007 | ouabain | ATPase inhibitor |
| -95.84 | cp | BRD-K03109492 | NSC-663284 | CDC inhibitor |
| -95.77 | cp | BRD-K24681473 | YM-155 | Survivin inhibitor |
| -95.74 | cp | BRD-A98283014 | calmidazolium | Calcium channel blocker |
| -95.74 | cp | BRD-A62025033 | temsirolimus | MTOR inhibitor |
| -95.61 | cp | BRD-U51951544 | ZG-10 | JNK inhibitor |
| -95.47 | cp | BRD-K12184916 | dactolisib | MTOR inhibitor |
| -95.22 | cp | BRD-K14618467 | IKK-16 | IKK inhibitor |
| -94.97 | cp | BRD-A36630025 | SN-38 | Topoisomerase inhibitor |
| -94.8 | cp | BRD-K12994359 | valdecoxib | Cyclooxygenase inhibitor |
| -94.68 | cp | BRD-K13390322 | AT-7519 | CDK inhibitor |
| -94.19 | cp | BRD-A81772229 | simvastatin | HMGCR inhibitor |
| -94.19 | cp | BRD-K69328504 | L-690488 | Inositol monophosphatase inhibitor |
| -94.16 | cp | BRD-A15079084 | phorbol-12-myristate-13-acetate | PKC activator |
| -94.04 | cp | BRD-K37798499 | etoposide | Topoisomerase inhibitor |
| -93.96 | cp | BRD-K69840642 | CAY10603 | HDAC inhibitor |
| -93.73 | cp | BRD-K78373679 | RO-3306 | CDK inhibitor |
| -93.71 | cp | BRD-K04887706 | AKT-inhibitor-1-2 | AKT inhibitor |
| -93.69 | cp | BRD-K26664453 | cytochalasin-b | Microtubule inhibitor |
| -93.59 | cp | BRD-K74761218 | WT-171 | HDAC inhibitor |
| -93.57 | cp | BRD-A45498368 | WYE-125132 | MTOR inhibitor |
| -93.56 | cp | BRD-A13133631 | fluorometholone | Glucocorticoid receptor agonist |
| -93.52 | cp | BRD-K13566078 | BMS-345541 | IKK inhibitor |
| -93.42 | cp | BRD-K77008974 | WYE-354 | MTOR inhibitor |
| -93.23 | cp | BRD-K83289131 | CAY-10618 | NAMPT inhibitor |
| -93.23 | cp | BRD-K64890080 | BI-2536 | PLK inhibitor |
| -93.18 | cp | BRD-A15010982 | HU-211 | Glutamate receptor antagonist |
| -92.74 | cp | BRD-K84595254 | strophanthidin | ATPase inhibitor |
| -92.67 | cp | BRD-K08219523 | 5-nonyloxytryptamine | Serotonin receptor agonist |
| -92.52 | cp | BRD-K13514097 | everolimus | MTOR inhibitor |
| -92.4 | cp | BRD-A63998256 | helveticoside | ATPase inhibitor |
| -92.28 | cp | BRD-K09499853 | KU-0060648 | DNA dependent protein kinase inhibitor |
| -92.11 | cp | BRD-K28907958 | CD-437 | Retinoid receptor agonist |
| -91.89 | cp | BRD-K78126613 | menadione | Mitochondrial DNA polymerase inhibitor |
| -91.76 | cp | BRD-A22713669 | BVT-948 | Tyrosine phosphatase inhibitor |
| -91.48 | cp | BRD-K28296557 | AKT-inhibitor-IV | AKT inhibitor |
| -91.41 | cp | BRD-A78360835 | cercosporin | Photoactivated toxin |
| -91.22 | cp | BRD-K29733039 | deforolimus | MTOR inhibitor |
| -91 | cp | BRD-K15563106 | phloretin | Sodium/glucose cotransporter inhibitor |
| -90.98 | cp | BRD-A52650764 | ingenol | PKC activator |
| -90.82 | cp | BRD-A69960130 | bromocriptine | Dopamine receptor agonist |
| -90.73 | cp | BRD-A19248578 | latrunculin-b | Actin polymerization inhibitor |
| -90.67 | cp | BRD-K70490179 | rimcazole | Sigma receptor antagonist |
| -90.6 | cp | BRD-K84895041 | BMY-45778 | IP1 prostacyclin receptor agonist |
| -90.46 | cp | BRD-A34205397 | suloctidil | Adrenergic receptor antagonist |
| -90.45 | cp | BRD-K05653692 | DL-PDMP | Glucosyltransferase inhibitor |
| -90.38 | cp | BRD-U37049823 | HG-6-64-01 | RAF inhibitor |
| -90.29 | cp | BRD-K28360340 | TW-37 | BCL inhibitor |
| -90.14 | cp | BRD-K25504083 | cytochalasin-d | Actin polymerization inhibitor |
| -89.95 | cp | BRD-K81418486 | vorinostat | HDAC inhibitor |
| -89.85 | cp | BRD-K17705806 | JTC-801 | Opioid receptor antagonist |
| -89.66 | cp | BRD-A72711497 | lasalocid | Bacterial permeability inducer |
| -89.57 | cp | BRD-A65767837 | hydrocortisone | Glucocorticoid receptor agonist |
| -89.57 | cp | BRD-K47983010 | BX-795 | IKK inhibitor |
| -89.55 | cp | BRD-A92439610 | triamcinolone | Glucocorticoid receptor agonist |
| -89.45 | cp | BRD-A89434049 | sarmentogenin | ATPase inhibitor |
| -89.34 | cp | BRD-K12867552 | THM-I-94 | HDAC inhibitor |
| -89.24 | cp | BRD-K77908580 | entinostat | HDAC inhibitor |
| -88.87 | cp | BRD-K24132293 | piperlongumine | Glutathione transferase inhibitor |
| -88.77 | cp | BRD-K13049116 | BMS-754807 | IGF-1 inhibitor |
| -88.57 | cp | BRD-K68174511 | torin-2 | MTOR inhibitor |
| -88.55 | cp | BRD-K70914287 | BIBX-1382 | EGFR inhibitor |
| -88.52 | cp | BRD-K73395020 | SA-1478088 | -666 |
| -88.49 | cp | BRD-A60245366 | AS-601245 | JNK inhibitor |
| -88.49 | cp | BRD-K31912990 | CGP-71683 | Neuropeptide receptor antagonist |
| -88.47 | cp | BRD-K67566344 | KU-0063794 | MTOR inhibitor |
| -88.35 | cp | BRD-K16406336 | methylene-blue | Guanylyl cyclase inhibitor |
| -88.32 | cp | BRD-K15600710 | obatoclax | BCL inhibitor |
| -88.28 | cp | BRD-K21806131 | tegaserod | Serotonin receptor partial agonist |
| -87.84 | cp | BRD-A19633847 | perhexiline | Carnitine palmitoyltransferase inhibitor |
| -87.81 | cp | BRD-U86922168 | QL-XII-47 | BTK inhibitor |
| -87.74 | cp | BRD-K82823804 | SA-792987 | PKC inhibitor |
| -87.64 | cp | BRD-K06750613 | GSK-1059615 | PI3K inhibitor |
| -87.64 | cp | BRD-K68873215 | phosphodiesterase-V-inhibitor-II | Phosphodiesterase inhibitor |
| -87.51 | cp | BRD-U25771771 | WZ-4-145 | EGFR inhibitor |
| -87.48 | cp | BRD-K22503835 | scriptaid | HDAC inhibitor |

Supplementary table：The list of top 100 small molecule compound with highest negative score predicted by using regulated genes between late stage DN and healthy controls.

| Score | Type | ID | Name | Description |
| --- | --- | --- | --- | --- |
| -98.41 | cp | BRD-K69840642 | ISOX | HDAC inhibitor |
| -98.06 | cp | BRD-K02130563 | panobinostat | HDAC inhibitor |
| -97.82 | cp | BRD-K13390322 | AT-7519 | CDK inhibitor |
| -97.6 | cp | BRD-K68202742 | trichostatin-a | HDAC inhibitor |
| -97.58 | cp | BRD-A79465854 | auranofin | NFkB pathway inhibitor |
| -97.25 | cp | BRD-A30437061 | camptothecin | Topoisomerase inhibitor |
| -97.15 | cp | BRD-K64606589 | apicidin | HDAC inhibitor |
| -96.97 | cp | BRD-A59985574 | topotecan | Topoisomerase inhibitor |
| -96.68 | cp | BRD-A39646320 | HC-toxin | HDAC inhibitor |
| -96.66 | cp | BRD-K22503835 | scriptaid | HDAC inhibitor |
| -96.37 | cp | BRD-K81418486 | vorinostat | HDAC inhibitor |
| -96.32 | cp | BRD-U51951544 | ZG-10 | JNK inhibitor |
| -96.26 | cp | BRD-A71459254 | cymarin | ATPase inhibitor |
| -95.64 | cp | BRD-K08554278 | bisbenzimide | DNA binding agent |
| -95.53 | cp | BRD-A36630025 | SN-38 | Topoisomerase inhibitor |
| -95.37 | cp | BRD-K03109492 | NSC-663284 | CDC inhibitor |
| -95.34 | cp | BRD-A85860691 | chaetocin | Histone lysine methyltransferase inhibitor |
| -95.33 | cp | BRD-A49765801 | fludroxycortide | Glucocorticoid receptor agonist |
| -95.14 | cp | BRD-K63606607 | bufalin | ATPase inhibitor |
| -95.03 | cp | BRD-K12867552 | THM-I-94 | HDAC inhibitor |
| -94.94 | cp | BRD-K74761218 | WT-171 | HDAC inhibitor |
| -94.91 | cp | BRD-K24681473 | YM-155 | Survivin inhibitor |
| -94.39 | cp | BRD-K82143716 | flucytosine | Antifungal |
| -94.36 | cp | BRD-A13133631 | fluorometholone | Glucocorticoid receptor agonist |
| -94.22 | cp | BRD-U82589721 | HG-5-113-01 | Protein kinase inhibitor |
| -93.79 | cp | BRD-K14618467 | IKK-16 | IKK inhibitor |
| -93.55 | cp | BRD-K06426971 | ryuvidine | Histone lysine methyltransferase inhibitor |
| -93.52 | cp | BRD-A02481876 | importazole | Importin-beta transport receptor inhibitor |
| -93.23 | cp | BRD-A94756469 | digoxin | ATPase inhibitor |
| -93.05 | cp | BRD-K78373679 | RO-3306 | CDK inhibitor |
| -92.99 | cp | BRD-K15108141 | gemcitabine | Ribonucleotide reductase inhibitor |
| -92.87 | cp | BRD-K33583600 | isoliquiritigenin | Guanylate cyclase activator |
| -92.79 | cp | BRD-A68930007 | ouabain | ATPase inhibitor |
| -92.63 | cp | BRD-K02965346 | SU-11274 | Hepatocyte growth factor receptor inhibitor |
| -92.6 | cp | BRD-K18518344 | digitoxigenin | ATPase inhibitor |
| -92.39 | cp | BRD-K15563106 | phloretin | Sodium/glucose cotransporter inhibitor |
| -92.32 | cp | BRD-K12994359 | valdecoxib | Cyclooxygenase inhibitor |
| -92.08 | cp | BRD-A45333398 | periplocymarin | Apoptosis stimulant |
| -92.02 | cp | BRD-A80502530 | cinobufagin | ATPase inhibitor |
| -91.68 | cp | BRD-K37798499 | etoposide | Topoisomerase inhibitor |
| -91.63 | cp | BRD-K17140735 | SCH-79797 | Proteasome inhibitor |
| -91.29 | cp | BRD-A93236127 | digitoxin | ATPase inhibitor |
| -90.7 | cp | BRD-A78360835 | cercosporin | Photoactivated toxin |
| -90.51 | cp | BRD-K10705233 | GW-405833 | Cannabinoid receptor agonist |
| -90.03 | cp | BRD-A34806832 | proscillaridin | ATPase inhibitor |
| -89.89 | cp | BRD-K04853698 | LDN-193189 | Serine/threonine kinase inhibitor |
| -89.88 | cp | BRD-K28907958 | CD-437 | Retinoid receptor agonist |
| -89.66 | cp | BRD-A65440446 | cimaterol | Adrenergic receptor agonist |
| -89.21 | cp | BRD-K84895041 | BMY-45778 | IP1 prostacyclin receptor agonist |
| -89.11 | cp | BRD-K15409150 | penfluridol | T-type calcium channel blocker |
| -88.96 | cp | BRD-K17743125 | belinostat | HDAC inhibitor |
| -88.51 | cp | BRD-A15010982 | HU-211 | Glutamate receptor antagonist |
| -88.39 | cp | BRD-K98490050 | amsacrine | Topoisomerase inhibitor |
| -88.17 | cp | BRD-A83326220 | brazilin | Nitric oxide production inhibitor |
| -88.15 | cp | BRD-K21565985 | xylazine | Adrenergic receptor agonist |
| -88.13 | cp | BRD-A62025033 | temsirolimus | MTOR inhibitor |
| -88.07 | cp | BRD-A22713669 | BVT-948 | Tyrosine phosphatase inhibitor |
| -88.02 | cp | BRD-A06352508 | SB-218078 | CHK inhibitor |
| -87.65 | cp | BRD-K83289131 | CAY-10618 | NAMPT inhibitor |
| -87.43 | cp | BRD-K92991072 | PAC-1 | Caspase activator |
| -87.06 | cp | BRD-K14441456 | tyrphostin-AG-556 | EGFR inhibitor |
| -86.71 | cp | BRD-K02526760 | QS-11 | ARFGAP inhibitor |
| -86.66 | cp | BRD-K13566078 | BMS-345541 | IKK inhibitor |
| -86.61 | cp | BRD-A72711497 | lasalocid | Bacterial permeability inducer |
| -86.57 | cp | BRD-K70490179 | rimcazole | Sigma receptor antagonist |
| -86.39 | cp | BRD-A34205397 | suloctidil | Adrenergic receptor antagonist |
| -84.76 | cp | BRD-A35588707 | teniposide | Topoisomerase inhibitor |
| -84.7 | cp | BRD-K59184148 | SB-216763 | Glycogen synthase kinase inhibitor |
| -84.3 | cp | BRD-K79090631 | CGP-60474 | CDK inhibitor |
| -84.21 | cp | BRD-K06543683 | bisindolylmaleimide-ix | CDK inhibitor |
| -84.06 | cp | BRD-K26664453 | cytochalasin-b | Microtubule inhibitor |
| -83.71 | cp | BRD-K19220233 | JNK-9L | JNK inhibitor |
| -83.17 | cp | BRD-A92439610 | triamcinolone | Glucocorticoid receptor agonist |
| -82.6 | cp | BRD-K00615600 | AG-14361 | PARP inhibitor |
| -81.67 | cp | BRD-A98283014 | calmidazolium | Calcium channel blocker |
| -81.27 | cp | BRD-K13810148 | givinostat | HDAC inhibitor |
| -80.46 | cp | BRD-A97739905 | ketoprofen | Cyclooxygenase inhibitor |
| -80.05 | cp | BRD-K17705806 | JTC-801 | Opioid receptor antagonist |
| -80.02 | cp | BRD-A08003242 | rhodomyrtoxin-b | sodium fluorescein uptake inhibitor |
| -79.98 | cp | BRD-K07762753 | aminopurvalanol-a | Tyrosine kinase inhibitor |
| -79 | cp | BRD-K15402119 | huperzine-a | Acetylcholinesterase inhibitor |
| -78.68 | cp | BRD-K47983010 | BX-795 | IKK inhibitor |
| -78.64 | cp | BRD-K15600710 | obatoclax | BCL inhibitor |
| -78.13 | cp | BRD-K84595254 | strophanthidin | ATPase inhibitor |
| -78.08 | cp | BRD-K43796186 | benzyl-quinazolin-4-yl-amine | EGFR inhibitor |
| -77.67 | cp | BRD-K28761384 | zuclopenthixol | Dopamine receptor antagonist |
| -77.52 | cp | BRD-K52522949 | NCH-51 | HDAC inhibitor |
| -76.75 | cp | BRD-A15079084 | phorbol-12-myristate-13-acetate | PKC activator |
| -76.5 | cp | BRD-K82823804 | SA-792987 | PKC inhibitor |
| -76.47 | cp | BRD-K59456551 | methotrexate | Dihydrofolate reductase inhibitor |
| -76.34 | cp | BRD-K56957086 | dacinostat | HDAC inhibitor |
| -76.13 | cp | BRD-A19248578 | latrunculin-b | Actin polymerization inhibitor |
| -75.95 | cp | BRD-K14791739 | fluticasone | Glucocorticoid receptor agonist |
| -75.46 | cp | BRD-K64890080 | BI-2536 | PLK inhibitor |
| -75.15 | cp | BRD-K08219523 | 5-nonyloxytryptamine | Serotonin receptor agonist |
| -75.01 | cp | BRD-K19554809 | MK-212 | Serotonin receptor agonist |
| -74.92 | cp | BRD-K05653692 | DL-PDMP | Glucosyltransferase inhibitor |
| -74.46 | cp | BRD-K28296557 | AKT-inhibitor-IV | AKT inhibitor |
| -74.03 | cp | BRD-K37312348 | kenpaullone | CDK inhibitor |
| -73.95 | cp | BRD-M16762496 | PIK-75 | DNA protein kinase inhibitor |
